# Structural and functional significance of *Aedes aegypti* AgBR1 flavivirus immunomodulator

**DOI:** 10.1128/jvi.01878-24

**Published:** 2025-04-24

**Authors:** Ane Martinez-Castillo, Diego Barriales, Mikel Azkargorta, Juan Diego Zalamea, Ana Ardá, Jesus Jimenez-Barbero, Monika Gonzalez-Lopez, Ana M. Aransay, Alejandro Marín-López, Erol Fikrig, Felix Elortza, Juan Anguita, Nicola G. A. Abrescia

**Affiliations:** 1 Structure and Cell Biology of Viruses Lab, Center for Cooperative Research in Biosciences (CIC bioGUNE) - Basque Research and Technology Alliance (BRTA) 73038, Derio, Spain; 2 Inflammation and Macrophage Plasticity Laboratory, CIC bioGUNE - BRTA 73038, Derio, Spain; 3 Proteomics Platform, CIC bioGUNE - BRTA 73038, Derio, Spain; 4 Chemical Glycobiology Laboratory, CIC bioGUNE - BRTA 73038, Derio, Spain; 5 IKERBASQUE, Basque Foundation for Science 197447, Bilbao, Spain; 6 Genome Analysis Platform, CIC bioGUNE - BRTA 73038, Derio, Spain; 7 Centro de Investigación Biomédica en Red de Enfermedades Hepáticas y Digestivas (CIBERehd), Instituto de Salud Carlos III 468625, Madrid, Spain; 8 Section of Infectious Diseases, Department of Internal Medicine, School of Medicine, Yale University 5755, New Haven, Connecticut, USA; Michigan State University, East Lansing, Michigan, USA

**Keywords:** crystal structure, immunomodulators, mosquito-borne disease, cellular pathways, Zika virus, mosquito salivary proteins

## Abstract

Zika virus (ZIKV), an arbovirus, relies on mosquitoes as vectors for its transmission. During blood feeding, mosquitoes inoculate saliva containing various proteins. Recently, AgBR1, an *Aedes aegypti* salivary gland protein, has gained attention for its immunomodulatory potential, along with another protein, called NeSt1. We have determined the crystal structure of AgBR1 at 1.2 Å resolution. Despite its chitinase-like fold, we demonstrated that AgBR1 does not bind to chitobiose or chitinhexaose, while a key mutation in the catalytic site abrogates enzymatic activity, suggesting that the protein’s function has been repurposed. Our study also shows that AgBR1 and NeSt1, when presented to murine primary macrophages, alter cellular pathways related to virus entry by endocytosis, immune response, and cell proliferation. AgBR1 (and NeSt1) do not directly bind to the Zika virus or modulate its replication. We propose that their immunomodulatory effects on Zika virus transmission are through regulation of host-cell response, a consequence of evolutionary cross talk and virus opportunism. These structural and functional insights are prerequisites for developing strategies to halt the spread of mosquito-borne disease.

**IMPORTANCE:**

Our study informs on the structural and functional significance of a mosquito salivary gland protein, AgBR1 (along with another protein called NeSt1), in the transmission of the Zika virus (ZIKV), a mosquito-borne virus that has caused global health concerns. By analyzing AgBR1’s three-dimensional structure in combination with cellular and interaction studies, we discovered that AgBR1 does not function like typical proteins in its family—it does not degrade sugars. However, we show that it primes immune cells in a way that could help the virus enter cells more easily but not by interacting with the virus or altering viral replication. This finding is significant because it reveals how mosquito proteins, repurposed by evolution, can influence virus transmission without the virus’s direct presence. Understanding how proteins like AgBR1 work could guide the development of new strategies to prevent Zika virus spread, with potential relevance for other mosquito-borne viruses.

## INTRODUCTION

Arthropod-borne diseases are a global health concern, causing around 17% of total infections according to the World Health Organization. Mosquitoes are responsible for the transmission of serious human pathogens including dengue virus (DENV), Zika virus (ZIKV), Chikungunya virus, and Plasmodium parasites. Viruses and bacteria contain proteins that modulate the host immune system and induce cell responses that favor the infection. However, pathogens are not the only organisms evolved to evade and manipulate host responses. The saliva of hematophagous arthropods contains pharmacologically active proteins that modify the bite site environment to facilitate feeding. *Aedes aegypti* mosquitoes express more than 120 salivary gland proteins, with 30%–40% of functions being unknown (1, 2). Some of the described properties of these proteins are anti-hemostatic, anti-inflammatory, and immunomodulatory (3–5). Studies have demonstrated that mosquito saliva can facilitate viral transmission and contribute to the development of arthropod-borne diseases, as in the case of *Aedes aegypti* venom allergen-1 (AaVA-1) that acts as a positive regulator of autophagy and LTRIN, which inhibits the transcription factor NF-kB and the production of inflammatory cytokines (4, 6).

Recently, two additional *Aedes aegypti* proteins, *A. aegypti* bacteria-responsive 1 (AgBR1) and neutrophil stimulating factor 1 (NeSt1), have been described as inductors of neutrophil recruitment at the bite site, enhancing the dissemination of the virus during the early infection of ZIKV (7, 8). Immunization against AgBR1 or NeSt1 reduces and delays ZIKV viremia peaks, and AgBR1 also delays West Nile virus (WNV) infection (9).

No structural information exists on AgBR1, but based on its primary amino acid sequence, AgBR1 belongs to the glycoside hydrolase 18 (GH18) family of chitinases (7). NeSt1 shares a ~63.5% sequence identity with LIPS-2, a labrum-interacting salivary protein from *Aedes albopictus* saliva, which has been recently solved by X-ray showing a novel fold (10). NeSt1 is encoded by the *LIPS* gene in *Aedes aegypti*—hereafter NeSt1 and aeLIPS-2 refer to the same protein. Despite the above available information, our understanding of the cellular signaling cascades activated by the uptake of AgBR1 and NeSt1 in the host as well as their potential for the development of antiviral therapies remains limited.

To gain insights into the functional mechanisms of AgBR1, we determined its structure by X-ray crystallography at 1.2 Å resolution and assessed whether its presence in Zika-infected murine bone-marrow-derived macrophage (BMDM) cells would modulate viral replication. Furthermore, we performed proteomics studies to unravel the protein–protein network induced in BMDM cells by both AgBR1 and NeSt1 proteins. The crystal structure of AgBR1 shows a triose-phosphate isomerase (TIM) barrel with eight α-helices surrounding eight β-strands, a fold conserved across the GH18 family. Mutations in the catalytic site indicate that AgBR1 lacks enzymatic activity. Meanwhile, the distinct distribution of charges on the molecular surface and the loss of binding to chitobiose and chitinhexaose oligosaccharides suggest that the conserved fold has been evolutionarily repurposed for a different function. We show that the presence of AgBR1 does not alter viral replication in infected BMDM cells. However, the mere presence of AgBR1 or NeSt1/aeLIPS-2 in mouse BMDM cells altered functional networks related to virus entry, cell survival and proliferation, and innate immune response regulation as identified by protein–protein association networks and ingenuity pathway analysis.

Our results offer insights into AgBR1, providing its three-dimensional structure at atomic detail offering a precise scaffold for the design of interactors or the mapping of specific interaction regions, and inform on the cellular pathways activated by the introduction of AgBR1 and NeSt1 in macrophages. This information is an essential prerequisite for developing therapeutics to reduce Zika virus burden in humans.

## RESULTS

### Polymorphism in the *AgBR1* gene sequence

AgBR1 was cloned from the salivary glands of the Ho Chi Minh (HCM) strain of *Aedes aegypti* mosquitoes. The cDNA sequence had a point mutation compared to the GenBank (XM_001660695.2) Liverpool strain (LVP) at the nucleotide site 695 A (LVP) to 522 G (HCM), resulting in a K190E mutation at the protein level. To verify this, a new batch of HCM salivary glands was dissected, and the AgBR1 gene was amplified, cloned, and sequenced. We showed that the HCM strain gene for AgBR1 has a missense mutation compared to the LVP strain. The new sequence has been deposited in GenBank (PP975153). This mutation is near the α4 helix and the disordered loop at residues R149-V170 (see below).

### AgBR1 sequence variation and core structural conservation suggest functional adaptation

**Fig 1:**
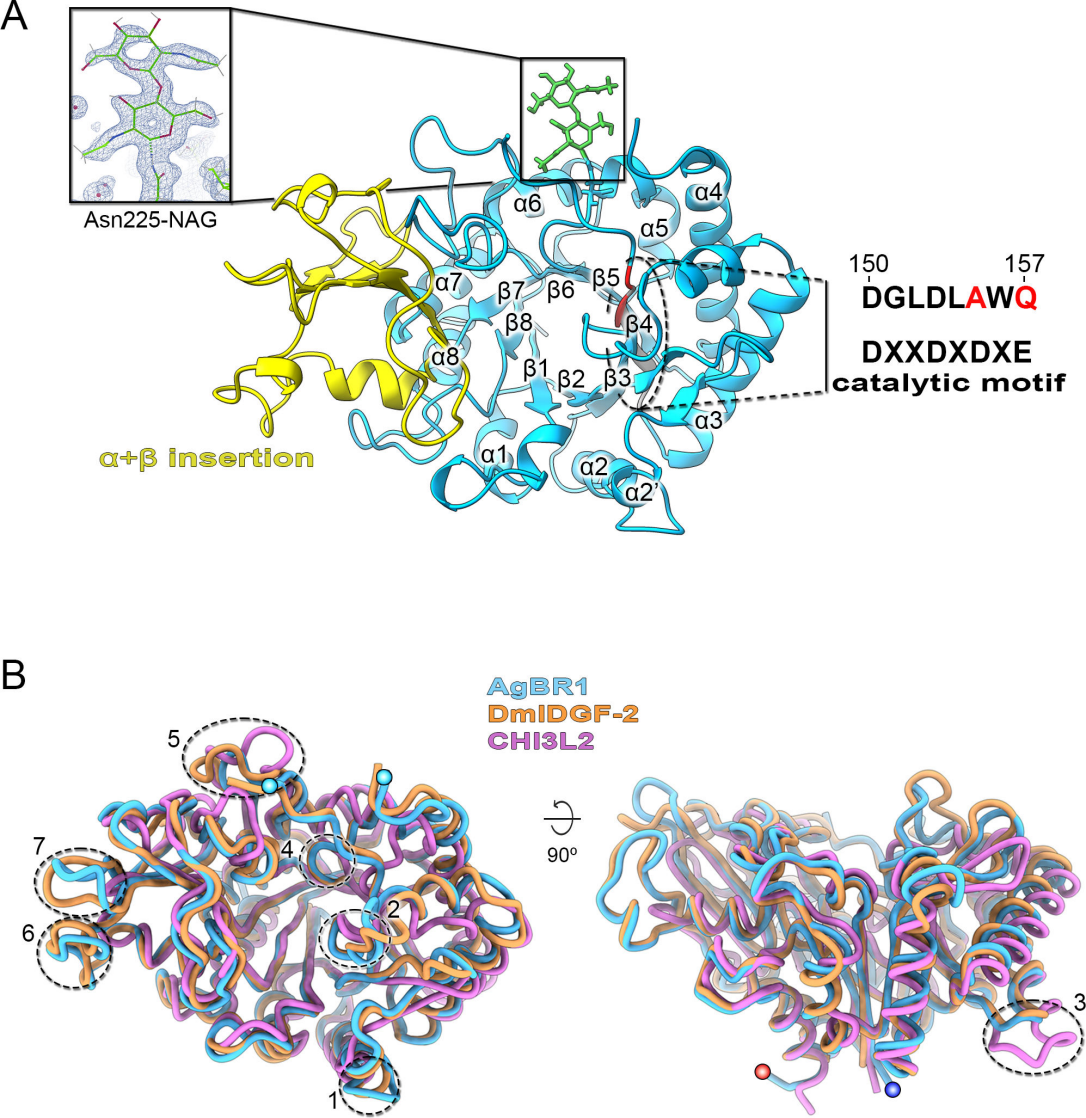
AgBR1 crystal structure and comparison with human and insect chitinase-like proteins. (A) AgBR1 crystal structure represented in cartoon containing β-sheets numbered from 1 to 8, surrounded by α-helices numbered from 1 to 8, colored in blue; in yellow, the α+β insertion domain between 285 and 375 residues. The black dashed oval indicates the putative catalytic site located on strand β4 (gray), with the residue leading to the abrogation of enzymatic activity in red. Right: the consensus sequence for catalytic activity with the sequence in AgBR1 on top. Left inset: the 2Fo–Fc density map contoured at the 1σ level of the two NAG sugar molecules attached to N225. (B) Structural superimposition of AgBR1 (light blue) onto insect DmIDGF-2 (orange) and human CHI3L2 (light magenta) represented as cartoon tubes. Cyan spheres in the left image indicate the last (R165) and first (V186) visible residues of the disordered loop, whereas the red and blue spheres in the right image mark the N- and C-terminal ends, respectively. Black numbered dashed circles mark some of the loops with different lengths and conformations (loop 1: L82-K86; loop 2: L109-P118; disordered loop: F158-E190; loop 3: V210-F214; loop 4: V220-V224; loop 5: Y266-N276; loop 6: P347-P360; loop 7: I378-G386).

The crystal structure of HCM AgBR1 was determined to 1.2 Å resolution, demonstrating AgBR1 to possess a glycoside hydrolase family 18 (GH18) chitinase fold. The core domain is a classical TIM barrel composed of eight parallel β-barrels (β1-β8) surrounded by eight α-helices (α1-α8) antiparallel to the barrel (Fig. 1A). The insertion domain formed by an additional α+β-fold between the β7 and α7 (residues Y302-Y391 compared to Imaginal Disc Growth Factor-2, IDGF-2) defines AgBR1 to belong to the subfamily A (11). The consensus motif of GH18 chitinases active site is DXXDXDXE, located in the β4 strand (12–14). In this region that expands from D150 to Q157, AgBR1 protein contains two mutations. The key conserved glutamic acid residue, acting as proton donor during hydrolysis of the glycoside bond, is substituted by Q (E157Q). In AgBR1, the D residue preceding Q157 in the motif, which stabilizes the catalytic amino acid and is part of the substrate-binding cleft, is substituted by A155 (Fig. 1A, right inset). These mutations lead to the loss of activity that catalyzes the hydrolysis of chitin. Like other GH18 family chitinases, it becomes a chitinase-like protein (CLP) without enzymatic function (15, 16). The achieved high resolution also resolves the only N-glycosylation site at the N207 residue, which shows two ordered N-acetylglucosamine (NAG) carbohydrates linked by a β(1-4) linkage, with the distal one being more flexible (Fig. 1A, left inset). Only one region of the AgBR1 molecule in the crystal structure (residues R165-V186), which corresponds to a loop between β4 and α4, displays weak electron density supporting a high degree of flexibility.

We then superimposed the AgBR1 structure onto two apo CLP (subfamily A) structures from the insect and human species, IDGF-2 (PDB id 1jnd) and human chitinase 3-like protein 2 (CHI3L2; PDB id 4p8u) (17, 18). These were selected because among all the 3D structures publicly available, they share the highest sequence identity with AgBR1, 52.6% and 30.4%, respectively (Fig. S1A). In terms of primary sequence, AgBR1 shares 99% sequence identity with other chitinases and CLPs in mosquito strains and up to 30% in humans, according to BLASTp analysis (https://blast.ncbi.nlm.nih.gov/Blast.cgi) (19, 20). The overall root-mean-square-deviation (rmsd) across the molecules’ backbone is relatively small, 1.09 Å across 386 superimposed Cα atoms with IDGF-2, and 1.97 Å across 335 superimposed Cα atoms with CHI3L2. However, the closest structural homolog to AgBR1 as derived from the Dali server is human chitinase 3-like protein 1 (CHI3L1), with an rmsd of 1.66 Å across 334 aligned residues and 29.6% of identity; practically CHI3L1 and CHI3L2 are equivalent structures (rmsd 1.12 Å across 318 residues) (Fig. S1B). Structural differences manifest mainly into different loop lengths and conformations. In AgBR1, among these loops are those connecting the structural motifs α2’-α2 (loop 1: L82-K86), β3-αa (loop 2: L109-P118), β4-α4 (disordered loop F158-E190), α4-β5 (loop 3: V210-F214; longer in CHI3L2), β5-αb (loop 4: V220-V224), the loop preceding α6 (loop 5: Y266-N276), and in the insertion domain, between αc-βc (loop 6: P347-P360) and βd-βe (loop 7: I378-G386) (Fig. 1B and Fig. S1A).

**Fig 2:**
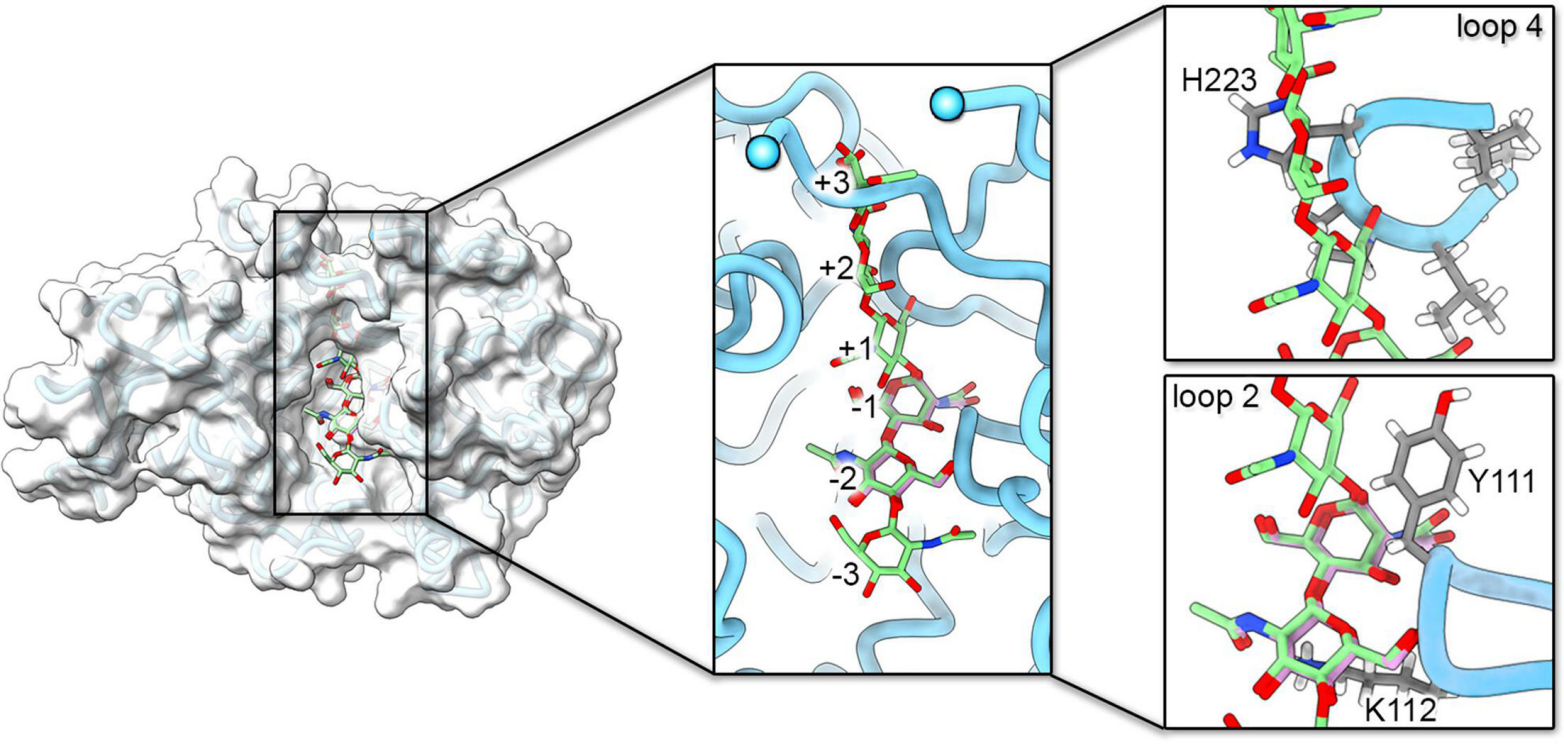
Composite of AgBR1 and sugar molecules. Surface representation of AgBR1 protein (semitransparent white) with the model depicted in light blue cartoon tube. Superimposed are GlcNAc6 (represented as a stick model, with oxygen and carbon atoms colored red and green, respectively) and GlcNAc2 (represented as a stick model, with oxygen and carbon atoms colored red and light magenta, respectively), derived from superimposition onto chitin-bound CHI3L2 structures (PDB ids 4p8x, 4p8v). Insets in the center and on the right show clashes at sugar positions -2 and -1 with loop 2, and at positions +1, +2, and +3 with loop 4 and residues preceding the disordered loop (R165-V186).

Superimposition with CHI3L2 with bound chitooligosaccharides (GlcNAc6 and GlcNAc2; PDB ids 4p8x, 4p8v) shows that AgBR1 residues G110, Y111, and K112 located between the β3-α3 loop, Q160, K162, and P163 in the loop β4-α4; L221, P222, and H223 in the loop β5-αb; and R254 in the loop between β6-α6 are responsible for the partial occlusion of the putative chitin-binding cleft. Y111 displays a double conformation, one of which forms a π–amide interaction with Q157, which in turn also stacks with Y123 (Fig. S2). The abovementioned AgBR1 residues are clashing with the CHI3L2 bound chitin oligosaccharide at positions -2, -1, +1, +2, and +3 (Fig. 2). To determine if AgBR1 binds chitin, we performed nuclear magnetic resonance-saturation transfer difference (NMR-STD) spectroscopy with chitobiose (GlcNAc2), the smallest of chitooligosaccharides. The STD spectrum showed no signals from the disaccharide (Fig. S3A). The same result was obtained for a longer chitin oligomer (GlcNAc6) (Fig. S3B). Because the STD effect relies on weak ligand binding (dissociation constant, K_D_, ranging from 10^−8^ to 10^3^ M) (21, 22), these results indicate that AgBR1 does not bind GlcNAc oligomers in this affinity range.

We further analyzed the charge distribution of AgBR1 to gain insights into its ability to interact with other substrates. The molecule displays a distinct separation of negative and positive charges on opposite sides (Fig. S4, left). On the side of the putative chitin-binding cleft, the major negatively charged patches are generated by residues E190, D192, E193, D196, and E197 on helix α4; by residues E309, D380, E381, E383, and E384 in loop 7 in the α+β insertion domain; and by residues E267, E270, and E273 in loop 2 next to β6 (Fig. S4 top left and Fig. S1A). A 180 degree rotation unravels a distinct positively charged hot spot on the surface, contributed by several residues. These include R165 in the C-terminal of β4, K257 in the loop between β6 and α6, and K310 in the insertion domain. Another positive region is formed by K26 in the N-terminal of β1, H58 in β2, K103 in the β3 region, and K430, R435, R440, K442, and H443 in α8. A third patch consists of K405 in the C-terminal of α7 and R369 in the α+β insertion domain between βc and βd (Fig. S4 bottom left and Fig. S1A). The charge segregation observed in AgBR1 contrasts with that of IDGF-2 and CHI3L2, where only one surface side appears to be predominantly negatively or positively charged in the latter and former cases, respectively (Fig. S4, center and right). Additionally, AgBR1’s putative binding cleft displays a neutral charge. Despite this, AgBR1 shares a set of conserved hydrophobic and negatively charged residues (Y30, R38, F63, Y111, F247, D248, Y301, F336, F415) with CHI3L2, which interact with chitooligosaccharides, comprising approximately 60% of the total interacting residues (18, 23). This conservation of residues in AgBR1, possibly a legacy of its chitooligosaccharide-binding role, is not sufficient for retaining the ability to bind chitobiose or chitinhexaose (Fig. 2).

### AgBR1 has no effect on virus replication in infected macrophages

Because AgBR1 and NeSt1 are released into the human host during the intradermal probing of the mosquito and the blood feeding phase along with ZIKV, we investigated whether AgBR1 (HCM strain) and NeSt1 (ORL strain) could directly interact with ZIKV (Uganda MR766 strain) ( 8, 9). Our enzyme-linked immunosorbent assay did not detect direct interaction with Zika virus (Fig. S5A). This finding was further supported for AgBR1 by nuclear magnetic resonance-heteronuclear single quantum coherence (NMR-HSQC) spectroscopy (Fig. S5B).

**Fig 3:**
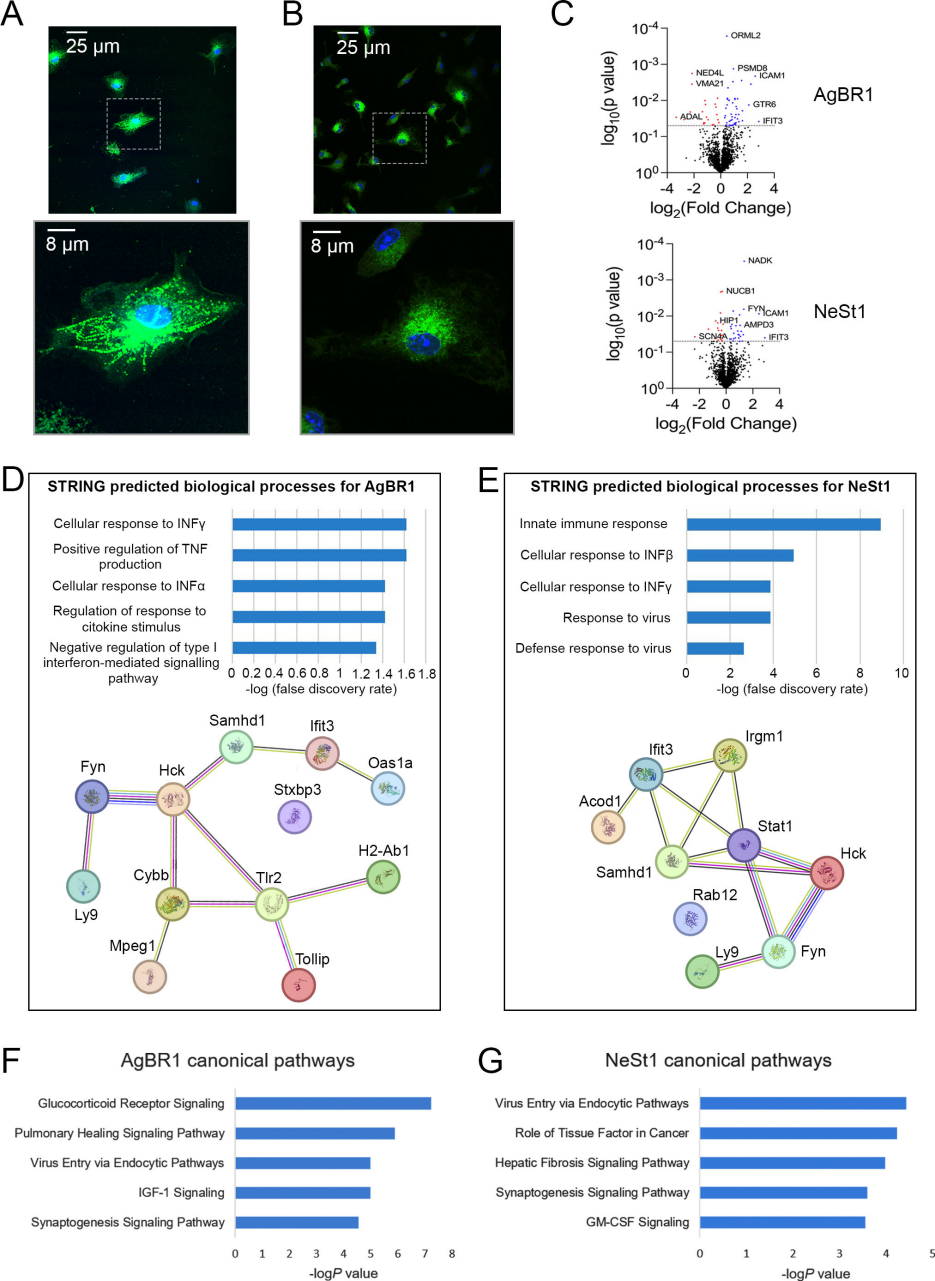
AgBR1 and NeSt1 effect on BMDM cells. BMDM cells incubated with 10 µg of Alexa-488-labeled AgBR1 (A) or NeSt1 (B) during 2 h at 37°C. The protein is internalized in both cases after incubation. (C) Volcano plot representing the proteins differentially expressed in BMDM cells in the presence or absence of AgBR1 and NeSt1. Blue and red dots represent upregulated and downregulated proteins above the significance threshold, respectively. (D and E) Innate immune response network extracted from STRING based on the BMDM over-/sub-expressed proteins in the presence of AgBR1 or NeSt1. The colored lines indicate the associations with different levels of certainty; green activation, red inhibition, blue binding, pink post-translation modified, black reaction, yellow expression, and gray evidence view. (F and G) Top five canonical pathways predicted by IPA for AgBR1 and NeSt1.

After the mosquito bites, flaviviruses encounter permissive cells for their replication (24). Monocytes, the primary target cells for ZIKV infection, serve as a “Trojan horse” aiding viral dissemination (25, 26). We incubated murine BMDM cells with Alexa-488-labeled AgBR1 and NeSt1. Each protein was internalized by BMDM cells within 2 hours. AgBR1 initially punctuated the cell surface (at 30 min, the fluorescence inside the cell was not apparent), with subsequent fluorescence showing endosomal trafficking to the perinuclear region (Fig. 3A). In contrast, NeSt1 fluorescence appeared more diffusely distributed, indicating transport from the cell periphery to the perinuclear region (Fig. 3B).

Next, to evaluate the influence of AgBR1 on cell viral load, we infected the BMDM cells with Zika virus in the presence or absence of AgBR1. Quantitative PCR (qPCR) analysis of viral RNA indicated no significant change in viral load when the protein was added, indicating that AgBR1 does not affect viral replication (Fig. S6; Table S1).

### AgBR1 and NeSt1 induce the activation of virus entry-related programs in macrophages

To assess the possible signaling pathways influenced by individual *Aedes* salivary gland proteins in macrophages, we compared their proteomes in the presence or absence of AgBR1 or NeSt1 after 24 hours of incubation. Protein expression levels were assessed using liquid chromatography mass spectrometry (LC-MS/MS), unraveling significant differences between the experimental conditions (Fig. 3C; Fig. S7A). A total of 55 proteins showed significant differential expression (*P* value < 0.05; fold changes ≤0.8 or ≥1.25) in response to AgBR1 stimulation, and 37 in response to NeSt1 stimulation (Tables S2 and S3). Fifteen proteins were commonly affected by both stimulations (Uniprot: Q8C878, Q3UV17, Q60710, P01899, Q01965, Q9JIW9, Q61549, Q9R233, P08103, P09671, P28667, P39688, P58058, P13597, Q64345) (Fig. S7B).

We used the STRING (Search Tool for the Retrieval of Interacting Genes/Proteins) database to analyze the significantly differentially expressed proteins identified as upregulated (fold change ≥1.25) and downregulated (fold change ≤0.8) in response to AgBR1 and NeSt1 (27). STRING analysis informed that proteins associated with AgBR1 were primarily linked to immune or defense responses. Notably, the “innate immune response” network showed a confidence level of 0.8 with a false discovery rate (FDR) of 0.025 (Table S4). This network involved biological processes such as “cellular response to interferons” (IFN-α), “positive regulation of tumor necrosis factor (TNF) production,” and “regulation of response to cytokine stimulus” (Fig. 3D; Table S5). For NeSt1 stimulation, STRING identified additional networks including “antigen processing and presentation,” “cellular response to interferons” (IFN-β), and “innate immune response” (Table S6). Within the “innate immune response” network, related biological processes included “cellular response to IFN-β,” “defense response to virus,” and “response to virus” (Fig. 3E; Table S7).

The proteomics-derived data were further analyzed using Ingenuity Pathway Analysis (IPA) to identify functional pathways (QIAGEN Inc., https://digitalinsights.qiagen.com/IPA) (28). IPA uncovered 170 canonical pathways associated with AgBR1 and 99 with NeSt1 in BMDM cells (Tables S8 and S9). Among the top 25 identified canonical pathways based on significance, AgBR1 was predicted to regulate (i) metabolism-related pathways, including NAD signaling pathway, mitochondrial dysfunction, IGF-1 signaling, and insulin receptor signaling; (ii) immune response pathways, such as IL-2, IL-4, antigen presentation pathway, and Th2 signaling pathway; and (iii) pathways related to cell survival and activation of myeloid cells via GM-CSF signaling and proliferation via the ERK5 signaling (Table S8). Of note, although not within the top 25, the phagosome formation process was identified as a possible activated pathway for AgBR1.

For NeSt1 stimulation, the top 25 significant canonical pathways included (i) immune responses such as IL-13 signaling, IL-15 production, IFN signaling, antigen presentation, and Th1/Th2 activation; (ii) processes related to cell membrane vesicle formation and trafficking like caveolar-mediated endocytosis, actin cytoskeleton, and phagosome formation; and (iii) cell activation, survival, and proliferation through GM-CSF, ERK/MAPK signaling, calpain protease regulation of cellular mechanics, and integrin signaling (Table S9). Significantly, IPA analysis highlighted “virus entry via endocytic pathways” among the top five enriched functional pathways for both AgBR1- and NeSt1-induced proteome changes (Fig. 3F and G).

According to the upstream regulators detected by IPA, 21 and 14 were inferred to be activated with a Z-score ≥ 2 by the presence of AgBR1 and NeSt1, respectively; all of them related to the immune system (Tables S8 and S9). In AgBR1 stimulation, most of them were cytokines including IL-27, IFN-α, IFN-β, IFN-γ, TNF, IL-1β, GM-CSF, IL-2, as well as NF-kB, TLR4, IFNAR, and FCGR2A. IL-6 appeared also activated (Z-score = 1.81). In the case of NeSt1, FCGR2A, IFN-α, IFN-β, IFN-γ, IFNAR, TNF, and STAT1 were inferred to be activated.

### Structural comparison of AgBR1 with representatives of glycoside hydrolase family 18

The conventional classification of chitinases relies on conserved sequences and domain architecture, including the catalytic domain, the α+β insertion domain (exclusively in subgroup A of GH18 chitinases), a serine-/threonine-rich domain, a carbohydrate-binding module (CBM), and a fibronectin type III-like domain (11, 29, 30).

To frame how AgBR1 structurally relates to other chitinases and CLPs, we pairwise compared AgBR1 with 13 selected crystal structures of the GH18 family and generated a structure-based phylogenetic tree (to date, using the query “Chitinase” AND “GH18 family” into the Protein Data Bank, ~553 X-ray structures can be retrieved). Our selection included apo-structure representatives across organisms. However, the archaeal GH18 chitinase structure lacks the CBM domain present in the sequence, and we also included a virally encoded ortholog.

**Fig 4:**
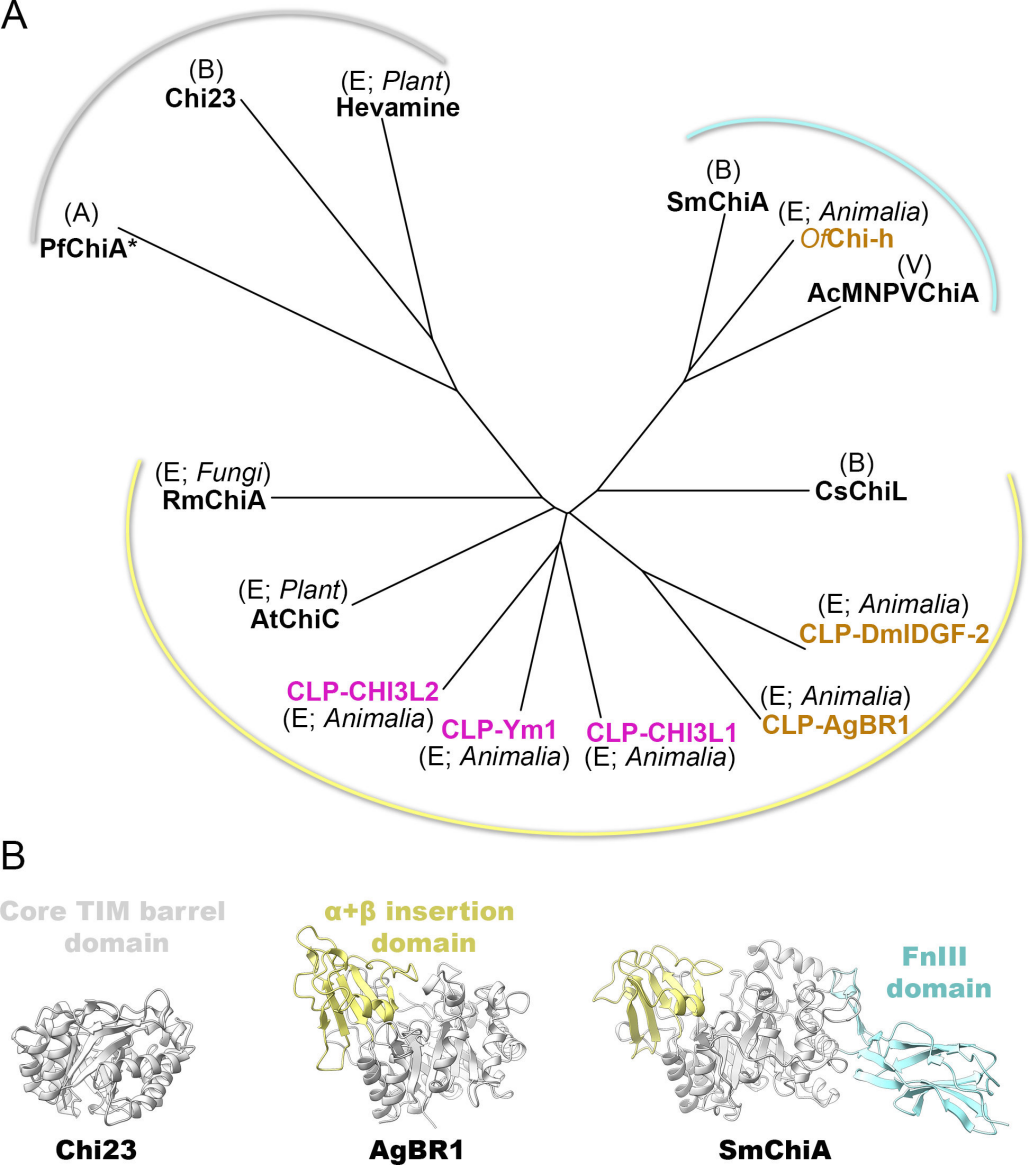
Structure-based superimposition of GH18 chitinase family proteins. (A) Structure-based phylogenetic tree of selected chitinases and chitinases-like of the GH18 family (*Drosophila melanogaster* Imaginal Disc Growth Factor-2 [DmIDGF-2], PDB id 1jnd; YKL-40-like protein 1 [Ym1], PDB id 1vf8; human chitinase 3-like protein 2 [CHI3L2], PDB id 4p8u; human chitinase 3-like protein 1 [CHI3L1], PDB id 1hjx; *Arabidopsis thaliana* Chitinase C [AtChiC], PDB id 3aqu; *Serratia marcescens* Chitinase A [SmChiA], PDB id 1ctn; *Autographa californica* Multiple Nucleopolyhedrovirus Chitinase A [AcMNPVChiA], PDB id 5dez; hevamine [from tree *Hevea brasiliensis*], PDB id 2hvm; *Pyrococcus furiosus* Chitinase A [PfChiA*, where the asterisk indicates the absence of the CBM domain in the structure, despite its presence in the sequence], PDB id 2dsk; *Pseudoalteromonas aurantia* Chi23, PDB id 6k7z; *Ostrinia furnacalis* [*Of*Chi-h], PDB id 5gpr; *Rhizomucor miehei* Chitinase 1 [RmChi1], PDB id 5xwf; *Chitiniphilus shinanonensis* [CsChiL], PDB id 6kst). The labels (A), (B), and (E) within the tree denote the kingdoms Archaea, Bacteria, Eukarya, respectively, and (V) stands for viruses, to which the chitinase and chitinase-like proteins (CLPs) belong. CLPs from mammals and insects are colored in magenta and gold, respectively. The tree displays three major clusters, each delineated by a curved line colored in gray, yellow, and cyan, representing the shared structural module depicted in (B). (B) Cartoon representation of the conserved structural fold (gray, left) and add-on motifs (yellow, center, and cyan, right) in chitinase and chitinase-like proteins.

The structure-based tree comprised three branches, with the rmsd across the Cα backbones ranging from 0.62 to 2.74 Å, confirming an overall structural conservation, which recapitulate the presence of “add-ons” subunits (Fig. 4A). One branch groups the simplest chitinases composed of only the TIM barrel catalytic domain: bacterial *Pseudoalteromonas aurantia* Chi23 and plant hevamine (archaeal *Pyrococcus furiosus* chitinase A [PfChiA] catalytic domain is nearby) (Fig. 4A and B, left). Although the TIM barrel domain is conserved across all chitinases and chitinase-like proteins within the family, structural variations are evident in the diverse conformations and lengths of loops connecting secondary structural elements. Most selected eukaryotic chitinases and chitinase-like proteins cluster together, sharing the α+β insertion domain apart from the core TIM barrel domain with CLPs belonging to the animal kingdom closer in structure than fungal *Rhizomucor miehei* Chitinase 1 (RmChi1) and plant *Arabidopsis thaliana* Chitinase C (AtChiC) nearby (Fig. 4A and B, center). Whether this difference might reflect minor structural variations related to their belonging to fungi and plant kingdoms remains to be seen (Fig. 4A). The insertion domain has been related to a deeper substrate-binding cleft for longer ligand binding (31, 32). The remaining cluster composed of the *Serratia marcescens* chitinase A (SmChiA), the baculovirus *Autographa californica* multiple nucleopolyhedrovirus chitinase A (AcMNPVChiA), and insect *Ostrinia furnacalis* chitinase-h represents those chitinases equipped with both the α+β insertion domain and the fibronectin type III domain (FnIII) (Fig. 4A and B, right).

In conclusion, while members of the GH18 chitinase family preserve their multi-domain architecture, they display structural variability in the loop regions. These regions are likely to undergo greater changes due to environmental pressure without compromising the stability of the overall fold. In AgBR1, these changes, along with mutations in the active site, have led to the repurposing of its potential original chitinase activity.

## DISCUSSION

Research on mosquito salivary gland extract is gaining more attention as the effect of some salivary proteins has been related to an increase in ZIKV and other flavivirus replication.

In our study, we targeted two salivary gland proteins *Aedes aegypti* AgBR1 (HCM strain) and NeSt1 (ORL strain). In the case of AgBR1, we elucidated its structure by X-ray crystallography to 1.2 Å resolution, demonstrating it to be a member of the GH18 family of chitinases. In contrast, the NeSt1 ortholog LIPS-2 from *Aedes albopictus* saliva was recently resolved, showing a novel fold (10). AgBR1 contains a key mutation, E157Q, in the active site consensus motif, leading to its loss of activity. Like other GH18 family chitinases, AgBR1 becomes a CLP without enzymatic function (15, 16). Furthermore, we identified a K190E mutation, resulting from a missense mutation at the nucleotide level of the Ho Chi Minh strain of mosquitoes compared to the Liverpool strain (LVP). This mutation represents a genetic polymorphism and is structurally located in a disordered loop. When superimposed with other eukaryotic CLPs, such as IDGF-2 (a growth factor that promotes cell proliferation in imaginal discs in *Drosophila melanogaster*) and human CHI3L2 (overexpressed in osteoarthritis, in glioblastoma, and in tumor-associated macrophages in breast cancer) (19, 20), AgBR1 showed a highly conserved core structure, while the most significant differences are found in the loops. This loop variability could account for differences in specificity and functionality. In fact, the superimposition of AgBR1 with the crystal structure of the CHI3L2-chitooligosaccharide complex indicates that its putative ligand-binding site is smaller and exhibits clashes with the ligand primarily caused by loops 2 and 4. Our NMR results show that neither chitobiose, a disaccharide consisting of two linked N-acetylglucosamine (GlcNAc) units, nor chitinhexaose, a hexasaccharide, interacts with the AgBR1 protein. This can be explained by the narrower putative chitin-binding site of AgBR1 compared to other chitin-binding CLPs (Fig. 2). The lack of binding of chitinhexaose strengthens the hypothesis that the binding of larger oligosaccharides is unlikely. In all cases, ligand binding in the accepted cleft would require the reorganization of three major structural motifs: loops 2 and 4, as well as the residues preceding the disordered loop—to widen the cleft (Fig. 2). For example, the estimated volumes of the binding site pockets for AgBR1 and DmIDGF-2 proteins are 511.5 and 297.4 Å³, respectively, smaller than those observed in CHI3L2 (639.5 Å³) or other chitinases such as bacterial SmChiA (1,012.7 Å³) (Fig. 2). Together with CHI3L2, other mammalian CLPs such as CHI3L1, SI-CLP, and Ym1 expressed mainly by immune cells (i.e., macrophages, neutrophils, dendritic cells), although inactive, retain the ability to bind carbohydrates (33). Nevertheless, studies on CHI3L1 have shown its capacity to bind to the IL-13Rα2 receptor independently of carbohydrate mediation (34, 35). The broad diversity of glycans in human target cells does not rule out, however, the possibility of AgBR1 interacting with other types of glycans but on different surface spots, as in the case of the human chitinase-like protein CHI3L1 binding to heparin (36). We also proved that neither AgBR1 nor NeSt1 interacts with ZIKV, in contrast to other *Aedes aegypti* salivary proteins (AAEL000793, AAEL007420, and AAEL006347), which bind to the virus envelope glycoprotein E with nanomolar affinities, and to orthologs of Cuticular Protein-19 (the binding partner of LIPS-2 in the *Aedes albopictus* labrum), which bind to WNV and dengue viruses (37, 38). These results suggest that both proteins are taken up by cells independently of any interaction with ZIKV, and we showed their internalization by BMDM cells after 2 hours of incubation. The presence of AgBR1 does not alter the replication of ZIKV in our targeted cells. This finding aligns with previous studies on other salivary gland proteins (e.g., AAEL000793, AAEL007420, and AAEL006347) performed in endothelial cells and keratinocytes (37). Similarly, the incubation of NeSt1 protein with human monocytic cell line (THP-1) did not alter the replication levels of ZIKV or DENV at 24 hours postinfection (hpi), but a recent study on skin explants demonstrated that, indeed, NeSt1 significantly increased Zika virus titers over a period of 24 to 96 hpi (6, 39). Other salivary gland proteins, such as AaVA-1, have been shown to also increase the replication of both ZIKV and DENV when incubated with monocyte-derived macrophages and dendritic cells (6).

While AgBR1 presence in BMDM cells does not alter viral replication, the simple presence of AgBR1 or NeSt1 does alter the proteome of the cells. In fact, STRING analysis that explores known and predicted protein–protein interactions associated these proteins with immune or defense responses. Additionally, IPA software identified “virus entry via endocytic pathways” as one of the top five most enriched canonical pathways.

The absence of the virus during the uptake of these proteins by BMDM cells suggests that AgBR1 and NeSt1 themselves may regulate this functional network, potentially through mechanisms such as receptor modulation or enhancement of endocytosis, among others. Interestingly, AgBR1 and NeSt1 could alter phagosome formation, while NeSt1 was also associated with caveolin-mediated endocytosis. Recent data have shown NeSt1 to reduce basal phagocytosis activity (39). Notably, we showed that both proteins were transported via the endocytic pathway in cellular experiments with labeled proteins, exhibiting different temporal dynamics. This suggests that AgBR1 and NeSt1 might enhance ZIKV internalization, in addition to the virus’s typical clathrin-mediated endocytosis mechanism. Although IPA predicted the IL-13 signaling pathway as being regulated by NeSt1 presence, when NeSt1 is used for stimulation experiments in human whole white blood cells, it does not influence or upregulate this pathway (39).

On the other hand, INF-γ and TNF are two upstream regulators predicted by IPA as being activated in the presence of AgBR1 or NeSt1. IL-6 is predicted to be activated only in the presence of AgBR1 and STAT1 and IL-1β only in presence of NeSt1. Data derived from stimulation of human whole white blood cells show that IFN-γ and TNF are inhibited, while transcriptomics of human skin sample indicate STAT1 to be repressed (39). In line with our results, studies show that NeSt1 and AgBR1 induced IL-1β in primary neutrophils and *in vivo*, respectively, while IL-6 was reported to be overexpressed in murine splenocytes in the presence of AgBR1 (7, 8). Additionally, IL-1β cytokine was also upregulated in human skin cuts at 4 hours *Aedes aegypti* post-bite (40).

Thus, our findings indicate that AgBR1 and NeSt1, on their own, regulate the production of numerous cytokines in BMDM cells when compared, for example, to the entire *Aedes aegypti* mosquito salivary gland extract. This aligns with studies on individual components of *Aedes aegypti* saliva that show various mechanisms by which they modulate immune responses in the skin and white blood cells (39, 40). Interestingly, exposure of human leukemia monocytic cells to *Aedes aegypti* salivary gland extract results in the downregulation of innate immune, antiviral, and inflammatory-related genes, including NF-kB, IFN-β, NLRP3, and subsequently IL-1β and IL-6, among others, at 24–36 hours (41). The example of AgBR1 and NeSt1 proteins demonstrates a synergistic effect leading to the regulation of innate immune responses and virus entry pathways as a global outcome.

**Fig 5:**
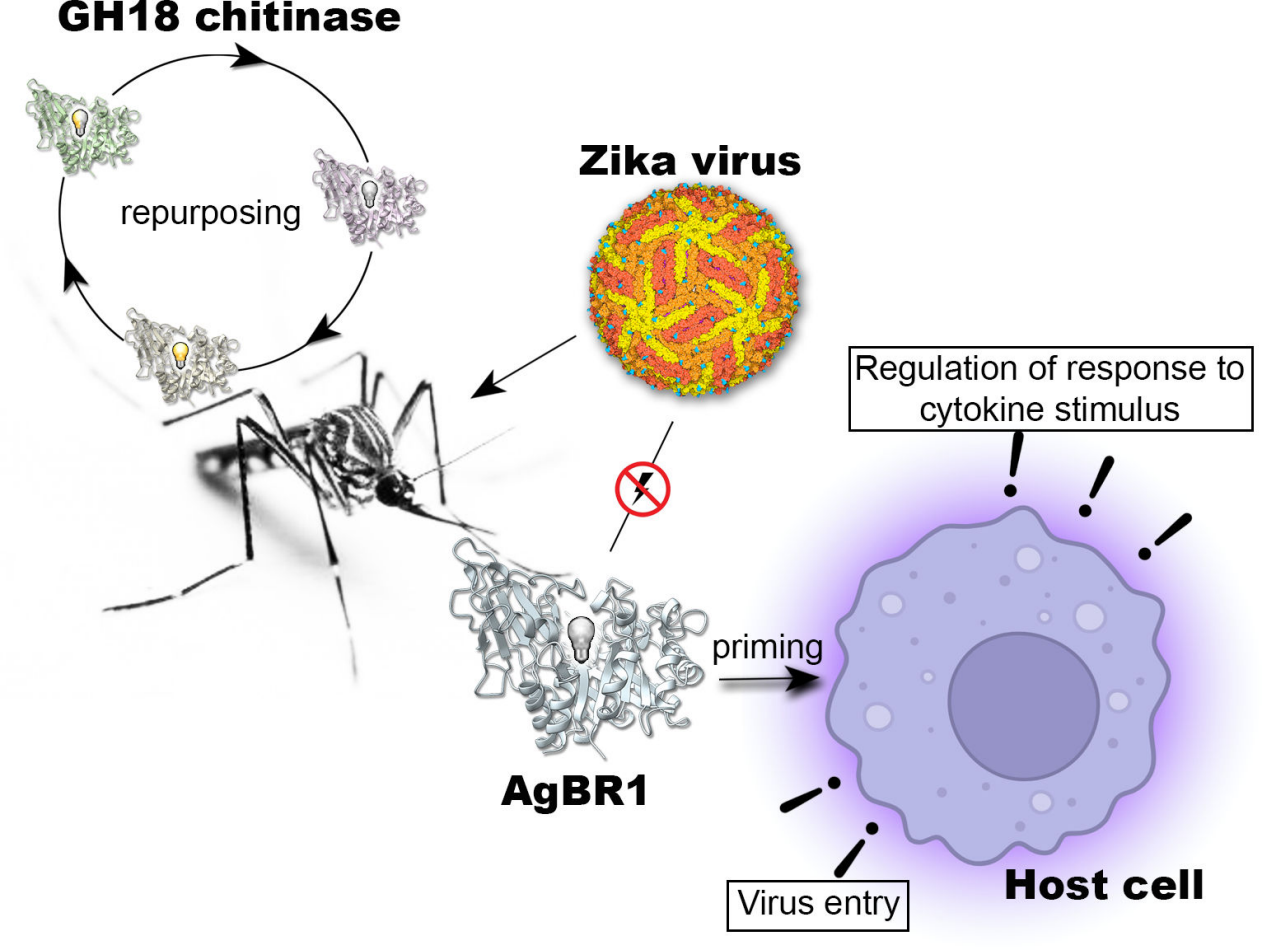
Putative adaptive function of AgBR1. Schematic illustrating the repurposing of a GH18-type chitinase with a common TIM barrel fold, known for binding and degrading chitin polymers (light on), into a structurally homologous protein that lacks both enzymatic and chitin-binding activity (light off); inactive but chitin binding (light half on). The repurposed mosquito salivary gland protein AgBR1 does not physically interact with the Zika virus or alter its replication, but it primes the immune response in murine macrophages by regulating viral entry through endocytotic pathways and influencing cytokine responses.

We propose that in *Aedes aegypti*, AgBR1 functions as a signaling protein when injected into human skin, based on its described structure and physicochemical properties. This is similar to other CLPs that bind cellular receptors without carbohydrate mediation (e.g., CHI3L1 binding to the IL-13Rα2 receptor) (34, 35, 42, 43). AgBR1 belongs to the family of the GH18 chitinases, and the provided structure-based phylogenetic tree of GH18 chitinases exemplifies how the differentially added domains across all organisms reflect paths of horizontal gene transfer and adaptation. Remarkably, the TIM barrel core emerges as an evolutionary robust and versatile protein scaffold that serves as a multifunctional platform onto which environmental pressures and evolutionary forces act (Fig. 5). *Aedes aegypti* possesses several functional chitinases essential for the mosquito’s life cycle, alongside chitinase-like proteins (44). While the former are involved in molting, cuticle formation, immune defense, and other physiological processes, the functions of the latter remain elusive. AgBR1 exemplifies nature’s adaptation of protein function through structural changes, including single mutations, loop variations, and domain insertions. In contrast, NeSt1 possesses a novel fold (as from its ortholog in *Aedes albopictus*) (10). Both salivary proteins interfere with the virus entry, immune, and cell proliferation pathways when priming murine macrophages. However, they do not directly bind to Zika virus or impact viral replication, nor do they possess enzymatic activity or the ability to bind chitobiose or chitinhexaose, as demonstrated for AgBR1 (Fig. 5).

We hypothesize that the evolutionary paths of these two salivary gland proteins, likely evolved to aid blood feeding, are exploited by Zika virus. Understanding 3D structures and cellular networks in this evolutionary cross talk provides insights for developing strategies to disrupt the transmission of mosquito-borne diseases.

## MATERIALS AND METHODS

### Cloning, recombinant expression, and purification of AgBR1 and NeSt1

*AgBR1* and *NeSt1* genes, initially in the pMT-Bip-V5-His tag vector (Invitrogen), were derived as previously described (7, 8). Prior to cloning into the pOPING vector (OPPF-UK) for large-scale recombinant protein mammalian expression, both genes were assessed by Sanger sequencing (STAB VIDA). The *AgBR1* gene from the HCM strain exhibited a single point mutation compared to the deposited corresponding gene from the Liverpool strain (GenBank XM_001660695.2); we confirmed this was a single nucleotide polymorphism, as detailed in the next section. The HCM *AgBR1* gene encoding for residues alanine at position 23 to leucine at position 441 was cloned into the pOPING vector using the In-Fusion cloning kit (Takara Bio). The final protein construct contained the extra residues ETG- and -KHHHHH at the N- and at the C-terminal ends, respectively, derived from the expression vector (see primers in Table S10). The sequenced *NeSt1* gene encoded nucleotide changes that led to amino acid mutations at E298A and N50D compared to the deposited Uniprot sequences Q17F11 (Liverpool strain) or Q8T9V1 (Black-eye strain). The E298A mutation was corrected during cloning to match the deposited Liverpool sequence while the N50D was left unchanged, as it was present in another deposited sequence of the Liverpool strain but with a deletion (sequence ID: EAT40400.1) and of a homologous protein LIPS-2 in *Aedes albopictus* (sequence ID: AAV90690.1) (see Table S10).

For recombinant protein production, ~5.5 × 10^7^ adherent HEK 293T cells were transfected using 1 mg of PEI MAX 4,000 (Polysciences) transfection agent and 0.5 mg of DNA construct per 2,125 cm^2^ roller bottle (Greiner). Transfection cocktail (50 mL) was prepared in Dulbecco’s modified Eagle medium (DMEM, Thermo Fisher) devoid of fetal bovine serum (FBS, Thermo Fisher). After 10 minutes of incubation, the media were added to a confluent roller bottle containing 200 mL of 2% FBS-DMEM media. The cells were incubated at 37°C and 5% CO_2_ for 1 week until the media were harvested for purification. HEK 293F cells were also transfected with 50 µg of plasmid and 50 µg of FectoPRO (Polyplus) transfection agent, in 10 mL of Freestyle 293 media per transfection cocktail. The mixture was incubated at room temperature for 10 minutes and loaded to a 10^6^ cells/mL confluent 0.5 L flask (Nunc) with 190 mL of media. The flasks were incubated for 5 days at 37°C and 130 rpm, when the media were harvested for purification.

Proteins were purified by nickel affinity chromatography using Ni-NTA Sepharose beads (GE Healthcare), followed by size exclusion chromatography using Superdex 75 (or 200) 10/300 GL column (GE Healthcare). First, the harvested media were centrifuged for 30 minutes at 6,000 × *g* and 4°C. The supernatant was filtered with 0.45 µm filters (Millipore), and then diluted four times in phosphate-buffered saline (PBS). Nickel-loaded Sepharose beads were added to the diluted media and incubated for 2 hours under mild shaking at 4°C. The beads were washed three times with 4 mL of 150 mM NaCl, 50 mM imidazole, and 50 mM Tris, pH 7.4 buffer. Each protein was eluted with 3 × 4 mL of 150 mM NaCl, 500 mM imidazole, and 50 mM Tris, pH 7.4 buffer. All eluted fractions (1 mL volume) were collected, and 10 µL of each was loaded onto a 10%–12% sodium dodecyl sulfate (SDS)-polyacrylamide gel electrophoresis (PAGE) gel. Fractions containing the targeted protein were combined and buffer exchanged using a 10 kDa cutoff Vivaspin500 (Sartorius) in 150 mM NaCl and 50 mM Tris, pH 7.4 buffer, then concentrated to 500 µL. The protein was loaded onto an S75 (or S200) column, and eluted fractions containing the protein were pooled and aliquoted. The aliquots were flash frozen with 10% glycerol and stored at −80°C.

### Validation of the point mutation in the *AgBR1* gene in the Ho Chi Minh strain

To validate the point mutation detected, we replicated the lifting of the gene from the mosquitoes as described elsewhere (7). Salivary glands from a pool of 30 *Aedes aegypti* mosquito of HCM strain were dissected and added to 100 µL of RLT buffer (QIAGEN ID 79216) . The mRNA was extracted obtaining a total amount of ~1,300 ng/µL. mRNA (15 µL) was retrotranscribed to cDNA, which was used for amplifying the *AgBR1* gene (no signal peptide). *AgBR1* was cloned with 5′ extended regions in the pEZT-BM vector (Addgene). The amplified insert was extracted from gel obtaining a total of 84 ng/µL in 20 µL. The vector was digested with *Xba* and *NotI* enzymes, and the resulting linear fragment was extracted from agarose gel. The cloning was performed using the In-Fusion Cloning kit (Takara Bio) with a 1:3 ratio of digested plasmid to insert. The sample was sequenced at Yale University sequencing platform and is deposited in GenBank with the code PP975153. Primers used in this study are listed in Table S10.

### AgBR1 crystallization and data collection

AgBR1 was screened for crystallization with PACT Premier, MidasPlus, and Proplex screens (Molecular Dimensions). Crystallization drops on a nanoliter scale were dispensed using a Mosquito robot (TTP LabTech) via the sitting-drop vapor-diffusion method into 96-well crystallization plates (MRC-2 and MRC-3). Thawed protein was desalted in 150 mM NaCl, 50 mM Tris, pH 7.4 buffer using Micro Bio-Spin columns (Bio-Rad) to eliminate glycerol. The absorbance of the concentrated AgBR1 used for crystallization as measured by nanodrop was ~8.7 mg/mL. Three protein:reservoir ratio conditions were tested for AgBR1: 100 nL of protein and 100 nL of reservoir buffer (1:1), 75 nL of protein and 150 nL of reservoir buffer (1:2), and 150 nL of protein and 75 nL of reservoir buffer (2:1). Crystals grew in the presence of 0.1 M MMT buffer, pH 4 and 25% PEG 1,500 at a ratio of 1:2 (protein:buffer). Crystals were fished out from the drop and flash frozen in 25% glycerol cryoprotectant. Diffraction data were collected on beamline I04 at Diamond Light Source (Didcot, UK) at a wavelength of 0.9795 Å and 100^o^K. A crystal belonging to space group *P*2_1_ with one molecule per asymmetric unit diffracted to 1.2 Å resolution. Collected data were automatically processed using the autoPROC/STARANISO pipeline at the beamline (45) (see Table 1).

**TABLE 1:** Data collection and refinement statistics of AgBR1

Variable	Result for AgBR1
Data collection	
X-ray source	I04 Diamond Light Source (UK)
Wavelength (Å)	0.97950
Space group	*P*2_1_
Cell dimensions	
*a*, *b*, *c* (Å)	50.43, 77.19, 51.12
α, β, γ (°)	90, 104.84, 90
Resolution (Å)	49.41–1.20 (1.33–1.20)^*a*^
R*merge*	0.044 (0.744)^*a*^
R*pim*	0.018 (0.355)^*a*^
*CC1/2*	0.999 (0.731)^*a*^
*I*/σ*I*	15.9 (1.53)^*a*^
Spherical completeness (%)	69.7 (13.8)^*a*^
Ellipsoidal completeness (%)	93.5 (51.3)^*a*^
Redundancy	6.68 (4.98)^*a*^
Measured reflections	542,557
Unique reflections	81,290
Refinement	
Data range (Å)	49.41–1.20 (1.25–1.20)^*a*^
No. reflections	81,286 (501)^*a*^
No. reflections *R-*free	3,991 (21)^*a*^
*R-*work	0.148 (0.236)^*a*^
*R-*free	0.18 (0.312)^*a*^
No. atoms	
Protein	6,410
Ligand/ion/carbohydrates	13/-/53
Water	332
*B*-factors (Å^2^)	
Protein	21.28
Ligand/ion/carbohydrates	72.48/-/66.08
Water	32.68
Ramachandran	
Most favored regions (%)	98.49
Allowed regions (%)	1.51
Disallowed regions (%)	0
Rotamer outliers (%)	0
Clashscore	1.7
rmsd deviations	
Bond lengths (Å)	0.007
Bond angles (°)	0.92

^*a*^ Values in parentheses are for the highest-resolution shell.

### AgBR1 crystal structure determination, refinement, and analysis

The structure of AgBR1 was solved by the molecular-replacement technique using, as a starting model, the crystal structure of Imaginal Disc Growth Factor-2 (PDB id 1jne; 54% sequence identity) in Phaser from CCP4 (46, 47). After manual model adjustment and visual inspection using the graphics software COOT, the model underwent refinement (48). The structure was initially rigid-body refined with the non-equivalent amino acids mutated to alanine and B-factors reset to 20 Å^2^ using the PHENIX software (49). Then, the refinement continued via gradient-based minimization using X-ray/stereochemistry weight (0.5), alternative conformation occupancy, and individual anisotropic B-factor refinement. Additional water molecules were added, ultimately resulting in a final model with a reliable geometry (Table 1).

The AgBR1 model was submitted to the DALI server (http://ekhidna2.biocenter.helsinki.fi/dali/), through which we selected two apo CLP (subtype A) structures: Imaginal Disk Growth Factor-2 (IDGF-2; PDB id 1jnd) and human chitinase 3-like protein 2 (CHI3L2; PDB id 4p8u) for cross-comparison. Analyses of the volume pockets of the different proteins were performed using CASTp software, while Chimerax was employed for the calculation of the isopotential surface (50, 51).

### Binding experiments by enzyme-linked immunosorbent assay (ELISA) and NMR spectroscopy

Prior to binding experiments ZIKV Uganda MR766 strain was propagated on Vero cells using a multiplicity of infection (MOI) of 1.0 and purified by 8% polyethylene glycol (PEG) 8000 precipitation, followed by a sucrose cushion at 24% and discontinuous K-tartrate gradient from 35% to 10% (5% intervals) as described elsewhere (52). The extracted fraction was buffer exchanged in NTE buffer (20 mM Tris, pH 8.0, 120 mM NaCl, 1 mM EDTA). The viral titer was determined using the TCID50 assay (53). Subsequently, wells of an ELISA plate (Nunc) were coated with 0.5 and 0.25 µg of AgBR1 or NeSt1 proteins per well in 100 mM carbonate/bicarbonate pH 9.6 buffer (total volume of 100 µL per well). Similarly, ZIKV was coated onto plates at approximately 200 viral particles per well. Coating was carried out by incubating the plates for 2 hours at room temperature. After coating, the wells were washed four times with 200 µL of PBS-Tween 20 (0.01%) (PBST) buffer between each step of incubation. Subsequently, the wells were blocked with 200 µL of 2% BSA-PBST buffer for 2 hours.

The binding was performed by adding ~10^3^ viral particles to the first well (in the case of wells containing protein) and 0.5 µg of protein in the first well (in the case of wells containing ZIKV), and then 1/2 serially diluted from 1 to 10. The plate was incubated for 1 hour at room temperature. Penta-His tag mouse monoclonal primary antibody (Thermo Fisher) was added at 1:1,000 dilution for wells coated with ZIKV, as the second layer was of protein. The same dilution was used for anti-E ZIKV mouse monoclonal primary antibody (clon ZV-54, MABF2046, Merck) to detect the virus in the wells coated with protein. The plate was incubated for 1 hour. The secondary antibody anti-mouse IgG, HRP conjugated, was added at 1:4,000 dilution and was incubated for 1 hour. The assay was developed using TMB substrate. Wells coated without any protein or virus but incubated against primary and secondary antibodies were used as negative control or blank. The positive control for protein was performed by coating the wells with a starting concentration of 0.5 µg of protein, and for the virus, ~10^3^ viral particles, 1/2 serially diluted from 1 to 10. The viral particles were previously incubated at 40°C for 10 minutes to un-envelope the virus.

For the detection of AgBR1 protein binding to Zika virus binding by NMR heteronuclear single quantum coherence (NMR-HSQC) spectroscopy, AgBR1 labeling was performed as follows. During the transfection of HEK 293F cells, 0.6 mg of 13C-glucose was added to 200 mL of 2% FBS DMEM media at a confluence of 10^6^ cells/mL. Five hundred microliters of 37 µM of purified protein was obtained and mixed in the presence of 10 µL of 10^6^ ZIKV Uganda MR766 strain particles. For the control sample preparation, AgBR1 at the same concentration was mixed with a 24% sucrose cushion purified from non-infected clarified Vero cells. NMR spectra were recorded on an 800 MHz Bruker AVANCE III spectrometer. ^1^H,^13^C-HSQC-NMR spectra were acquired with the constant time (CT) version, at 308 K, with 96 Hz resolution in F1 and 24 number of scans. ^1^H,STD-NMR spectra were acquired at 298 K, with 1,024 number of scans. On-resonance saturation was achieved by PC9 selective pulses (90 Hz bandwidth) at 0.39 ppm with a total saturation time of 2 seconds. A protein T1rho filter (40 ms) was employed.

To assess AgBR1 binding to chitobiose (a kind gift from Jindřich Karban and Martin Kurfiřt) and to chitinhexaose (Product code: GLU436-90%; ELICITYL) by NMR saturation transfer difference (STD-NMR), the AgBR1 protein was buffer exchanged into PBS, pH 7.4, prepared in deuterated water. AgBR1, 180 µL at 51 µM, was subjected to spectral acquisition both alone and in the presence of 80 equivalents of β-O-Me-chitobioside or chitinhexaose in 5 mm Shigemi NMR tubes and at 310 K. The spectra were acquired using the abovementioned 800 MHz Bruker spectrometer equipped with a TCI cryoprobe with z-gradient coil, and data were processed using TopSpin 3.2.7 (BRUKER) software. A total of 3,942 scans were collected, employing a Gauss-shaped pulse of 50 ms and 50 dB, with −1 and 100 ppm for the on and off resonance irradiation, respectively.

### Mice

C57Bl/6 (B6) mice were purchased from Charles River Laboratories (Lyon, France) and were bred in the Animal Facility at CIC bioGUNE, where they were socially housed under a 12 hour light/dark cycle.

### BMDM extraction

Murine BMDMs were extracted from the femoral and tibial shafts and were flushed twice throughout a 25G syringe with 6 mL of DMEM supplemented with 10% FBS and 1% penicillin/streptomycin. The content was then filtered with a tissue filter of 70 µm pore, centrifuged for 5 min at 300 × *g* and 4°C, and subjected to erythrocyte lysis with ACK Buffer (NH4Cl 150 mM, KHCO3 10 mM, Na2EDTA 0.1 mM). The cells were incubated in non-treated 100 mm × 15 mm plates (Thermo Fisher Scientific) for 6 days at 37°C in DMEM (Thermo Fisher Scientific) supplemented with 10% FBS and 1% penicillin/streptomycin plus 30 ng/mL of M-CSF (Miltenyi Biotec). The medium was changed every 3 days. At day 6, nonadherent cells were discarded, and differentiated BMDMs were scraped for experiments.

### AgBR1 and NeSt1 immunofluorescence microscopy

AgBR1 and NeSt1 entry assay was performed in BMDM cells. Purified proteins were labeled with Alexa Fluor 488 protein labeling kit (N^o^ A10235; Thermo Fisher) following the manufacturer’s instruction. Between 8.6 and 15.3 µM of labeled protein was obtained using 1.6–2.16 moles of dye per mole of protein.

BMDM cells (10^6^) per well were seeded in 6-well plates (Nunc) containing coverslips, and after overnight incubation, the cells were washed once with 2 mL of PBS. Cells were then incubated with 1 mL of media containing 10 µg of labeled AgBR1 or NeSt1 for 2 hours. The cells were subsequently washed twice with 2 mL PBS and fixed with 1 mL of 4% glutaraldehyde (Merck) for 10 minutes. After incubation, the cells were washed three times with 2 mL PBS and incubated with 50 µL of DAPI for nuclei staining at 1 mg/mL (Thermo Fisher). The cells were washed three times with 2 mL of PBS and loaded onto slides with 50 µL of Fluoromont (Thermo Fisher). Z-stack images were collected using the Leica TCS SP8 confocal microscope between 0.2 and 0.3 µm of thickness.

### Quantitative PCR analysis of ZIKV viral replication in infected BMDM cells in the presence of AgBR1

For qPCR experiments, 1 mL of BMDM cells was seeded in a 6-well plate at a concentration of 10^6^ cells/mL. Cells were infected with ZIKV (MR766 Uganda strain) at 0.1 or 0.5 MOI, and 5 µg of HCM AgBR1 protein diluted in PBS or PBS alone was added. Twenty-four hours after treatment, cells were collected, and RNA was extracted (RNeasy mini kit, QIAGEN). After RNA concentration determination by Qubit (Invitrogen) and RNA integrity assessment by Bioanalyzer (Agilent Technologies), 250–500 ng of RNA was retrotranscribed in a 50 µL reaction using M-MLV_Reverse Transcriptase (Invitrogen) and Random Hexamer Primers (Thermo Fisher Scientific). qPCR assays were conducted at the Genome Analysis Platform at CIC bioGUNE. Each 10 µL reaction was run in triplicate in a QuantStudio 5 System (Thermo Fisher) (2.5 minutes at 95°C; 40 cycles of 15 seconds at 95°C, 1 minute at 60°C; Melt Curve) with PerfeCTa SYBR Green SuperMix Low ROX (Quantabio), using 10 ng of cDNA as a template and primers at a final concentration of 1 µM. For the primer design of ZIKV MR766 Uganda strain *envelope glycoprotein E* gene, a multiple sequence alignment of GenBank ZIKV complete genome codes MK105975.1, KU955593.1, MH675629.1, KJ776791.2, and KU497555.1 was performed to secure the most conserved region as a forward primer. *Ribosomal Protein L-19* (*Rpl19*) and *Glyceraldehyde-3-phosphate dehydrogenase* (*Gapdh*) were used independently as reference genes (primer sequences listed on Table S1). Two independent experiments, using four biological replicates per experiment (BMDM cells of four different mice), were carried out (Fig. S5).

For data analysis, the ∆∆*Ct* method was used (54). Paired *t*-tests were calculated using GraphPad Prism 10.0.2 Software (Boston, MA, USA, www.graphpad.com).

### *In vitro* stimulation of BMDM cells with AgBR1 and NeSt1

BMDM cells (10^6^) per well were seeded in 6-well plates and maintained at 37°C and 5% CO_2_. After an overnight incubation, cells were incubated with 10 µg of protein per well (10 µg/mL). The purified protein was previously buffer exchanged in PBS. Under BSL-2 conditions, the protein was filtered through a 0.22 µm size pore filter (Millipore). After 24 hours of incubation with the protein, cells were washed three times with PBS and lifted mechanically. The control conditions were incubated with the same volume of PBS used during the buffer exchange process. The experiment was performed using BMDM from four independent mice.

### Proteomic analysis

Proteins were extracted from cells using a mixture of 7 M urea, 2 M thiourea, 4% CHAPS, and 200 mM DTT, and then digested following the filter-aided FASP protocol described elsewhere with minor modifications (55). Samples were analyzed on a timsTOF Pro with PASEF (Bruker Daltonics) coupled online to an Evosep ONE liquid chromatograph (Evosep). A total of 200 ng was directly loaded onto the Evosep ONE and resolved using the 30 sample-per-day protocol. Protein identification and quantification were carried out using MaxQuant software using default settings except for the match between runs (match time window of 5 minutes, alignment tie window of 20 minutes) and a label-free quantification (LFQ) minimum ratio count of 1 (56). Searches were carried out against a database consisting of mouse protein entries (Uniprot/Swissprot), with precursor and fragment tolerances of 20 ppm and 0.05 Da. Only proteins identified with at least two peptides at FDR < 1% were considered for further analysis. LFQ intensities were used for quantification analyses and loaded onto the Perseus platform for further statistical analysis (57).

### Analysis of identified proteins from cell stimulation experiments

Quantitative protein data were processed using Perseus software, a freely available tool developed by the Max Planck Institute, Munich, Germany. Differential protein abundance analyses were conducted within the program. Initially, protein abundance data underwent log2 transformation, filtered based on reproducibility criteria (retaining the proteins present in at least 70% of samples within a group), and imputed (missing values for the proteins that were not present in one of the conditions were substituted with abundances randomly taken from the 10% least abundant proteins in each sample). A Student’s *t*-test was used, whereby proteins exhibiting a *P* value less than 0.05 were considered significantly differentially expressed.

IPA software (QIAGEN) was used to identify pathways or regulators related to the differential protein patterns under analysis.

Furthermore, the STRING (https://string-db.org/) was used to identify protein–protein interactions and functional associations among the over-/underexpressed list of cellular proteins emerging by the uptake of AgBR1 and NeSt1. The selection of the proteins was based on the following criteria: (i) at least two identified peptides per protein by MS, (ii) a Student *t*-test of *P* < 0.05 between the two conditions (presence or absence of AgBR1 or NeSt1), and (iii) a fold change of >1.25 for the overexpressed proteins and <0.8 for the underexpressed ones.

The list of proteins over-/underexpressed by the presence of AgBR1 or NeSt1 is available in Tables S2 and S3.

### Structure-based phylogenetic tree

Structures of the GH18 family chitinase (DmIDGF-2, PDB id 1jnd; Ym1, PDB id 1vf8; CHI3L1, PDB id 1hjx; CHI3L2, PDB id 4p8u; AtChiC, PDB id 3aqu; SmChiA, PDB id 1ctn; AcMNPVChiA, PDB id 5dez; hevamine, PDB id 2hvm; PfChiA, PDB id 2dsk; Chi23, PDB id 6k7z; *Of*Chi-h, PDB id 5gpr; RmChi1, PDB id 5xwf; CsChiL, PDB id 6kst) were initially superimposed onto the AgBR1 structure using the secondary structure matching (SSM) routine in COOT and visually inspected (48). Then, the atomic models were used as input for the calculation of evolutive distances by Structure Homology Program (SHP) (58). A matrix with the values was generated with Fitch that was used to build the phylogenetic tree with the online Fitch (PHYLIP) T-REX webserver (59, 60). The representation of the tree was created with the online iTOL server (https://itol.embl.de/).

## Data Availability

Structure factors and atomic coordinates for AgBR1 structure have been deposited to the Protein Data Bank (http://www.wwpdb.org) with ID code 9G3Q. The mass spectrometry proteomics data have been deposited to the ProteomeXchange Consortium via the PRIDE (61) partner repository with the data set identifier PXD052671.

## SUPPLEMENTAL MATERIAL

Supplemental material jvi.01878-24-s0001.pdf Figures S1 to S7; legends for Tables S1 to S10.

Table S1 jvi.01878-24-s0002.xlsx qPCR primer sequences, data, and analysis.

Table S2 jvi.01878-24-s0003.xlsx Uniprot accession codes of the over-/underexpressed *Mus musculus* proteins in BMDM cells after 24 hours of incubation with AgBR1.

Table S3 jvi.01878-24-s0004.xlsx Uniprot accession codes of the over-/underexpressed *Mus musculus* proteins in BMDM cells after 24 hours of incubation with NeSt1.

Table S4 jvi.01878-24-s0005.xlsx Biological processes identified by STRING for AgBR1 differentially expressed proteins in BMDM cells.

Table S5 jvi.01878-24-s0006.xlsx Biological processes identified by STRING for AgBR1 inside the predicted “innate immune response” biological process.

Table S6 jvi.01878-24-s0007.xlsx Biological function processes identified by STRING for NeSt1 differentially expressed proteins in BMDM cells.

Table S7 jvi.01878-24-s0008.xlsx Biological processes identified by STRING for NeSt1 within the predicted “innate immune response” biological process.

Table S8 jvi.01878-24-s0009.xlsx Upstream regulators predicted by IPA for AgBR1 stimulation of BMDM cells.

Tables S9 and S10 jvi.01878-24-s0010.xlsx Table S9, canonical pathways predicted by IPA for NeSt1 stimulation of BMDM cells; Table S10, designed primers for AgBR1 and NeSt1 cloning and sequencing (5′-3′ direction).

ASM does not own the copyrights to Supplemental Material that may be linked to, or accessed through, an article. The authors have granted ASM a non-exclusive, world-wide license to publish the Supplemental Material files. Please contact the corresponding author directly for reuse.
