# Supplementary material for: Structural and functional significance of Aedes aegypti AgBR1 flavivirus immunomodulator
Source: J Virol. 2025 Apr 24;99(5):e01878-24. doi: 10.1128/jvi.01878-24 (PMC12090808; doi:10.1128/jvi.01878-24)
Supplement: Supplemental material — Figures S1 to S7; legends for Tables S1 to S10. [file jvi.01878-24-s0001.pdf]

SUPPORTING INFORMATION

The structural and functional significance of *Aedes aegypti* AgBR1 flavivirus immunomodulator

Ane Martinez-Castillo[1], Diego Barriales[2], Mikel Azkargorta[3], Juan Diego Zalamea[1], Ana Ardá[4,5], Jesus Jimenez-Barbero[4,5], Monika Gonzalez-Lopez[6], Ana M Aransay[6,8], Alejandro Marín-López[7], Erol Fikrig[7], Felix Elortza[3,8], Juan Anguita[2,5], Nicola GA Abrescia[1,5,8]

[1]Structure and Cell Biology of Viruses Lab, Center for Cooperative Research in Biosciences (CIC bioGUNE) - Basque Research and Technology Alliance (BRTA); Derio, Spain.

[2]Inflammation and Macrophage Plasticity Laboratory, CIC bioGUNE – BRTA; Derio, Spain.

[3]Proteomics Platform, CIC bioGUNE - BRTA; Derio, Spain.

[4]Chemical Glycobiology Laboratory, CIC bioGUNE – BRTA; Derio, Spain.

[5]IKERBASQUE, Basque Foundation for Science; Bilbao, Spain.

[6]Genome Analysis Platform, CIC bioGUNE – BRTA; Derio, Spain.

[7]Section of Infectious Diseases, Department of Internal Medicine, School of Medicine, Yale University, New Haven, CT, USA.

[8]Centro de Investigación Biomédica en Red de Enfermedades Hepáticas y Digestivas (CIBERehd), Instituto de Salud Carlos III; Madrid, Spain.

Correspondence: nabrescia@cicbiogune.es

Keywords: Zika virus, mosquito salivary proteins, immunomodulators

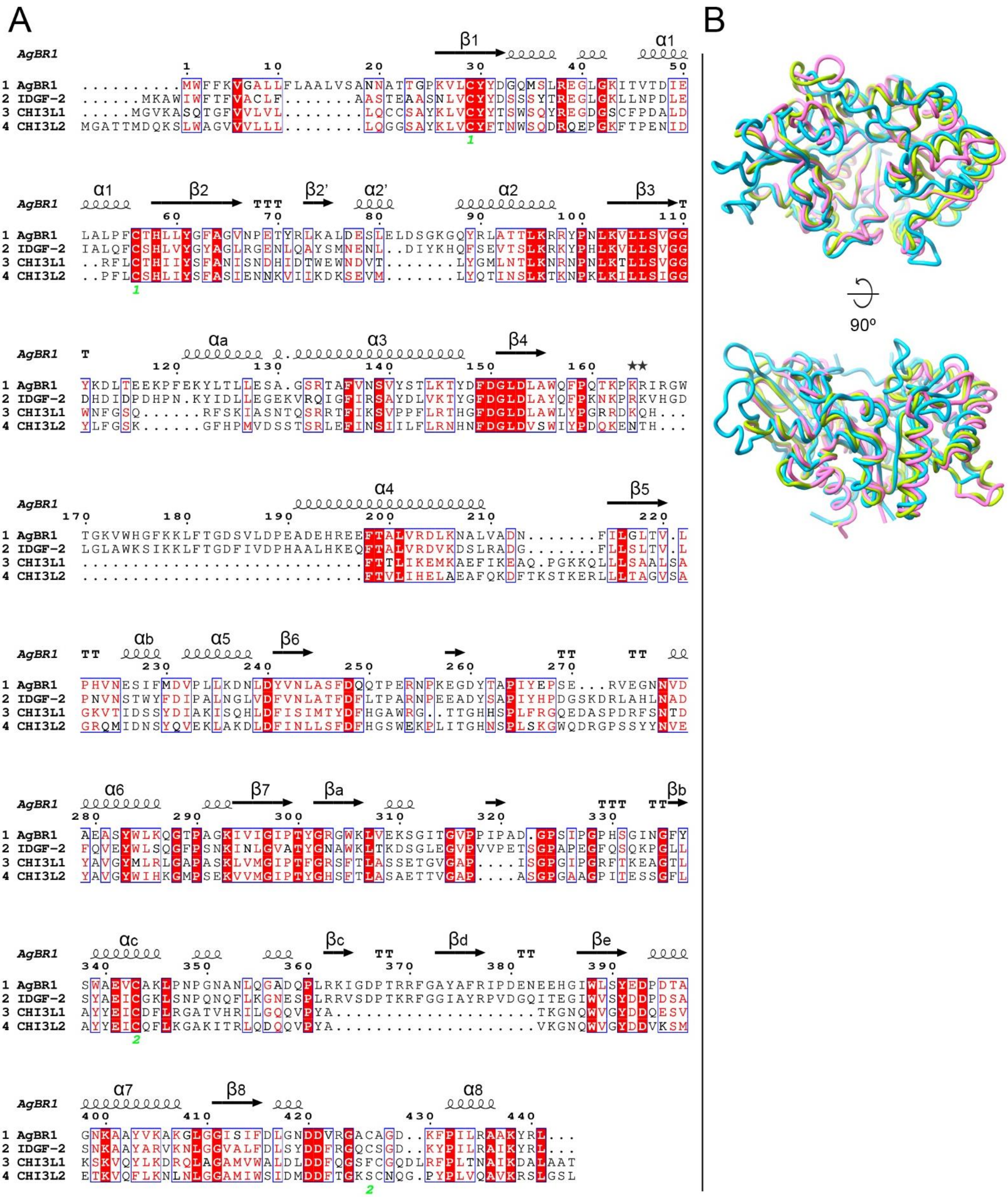

Supplementary Figure 1. (**A**) Sequence alignment between *Aedes aegypti* AgBR1, insect DmIDGF-2 and human CHI3L1 and CHI3L2 proteins, and secondary structure element derived using the solved 3D structure of AgBR1 and represented in the program Espript (1). Conserved residues are highlighted in red, while semi-conserved residues are colored in red.

The assignment of secondary structure nomenclature follows Varela et al. (2); helices and strands in the TIM barrel are numbered from 1 to 8 (apostrophe when discontinuous), while those outside the barrel are labeled with letters from a to e. (**B**) Structural superimposition of AgBR1 (cyan), CHI3L1 (lime), and CHI3L2 (light magenta) shows overall structural equivalence between CHI3L1 and CHI3L2 (and AgBR1), with minor differences in some connecting loops and in the region between β3 and α3, where CHI3L1 (and AgBR1) form a more structured α-helix and (see also Fig 1B).

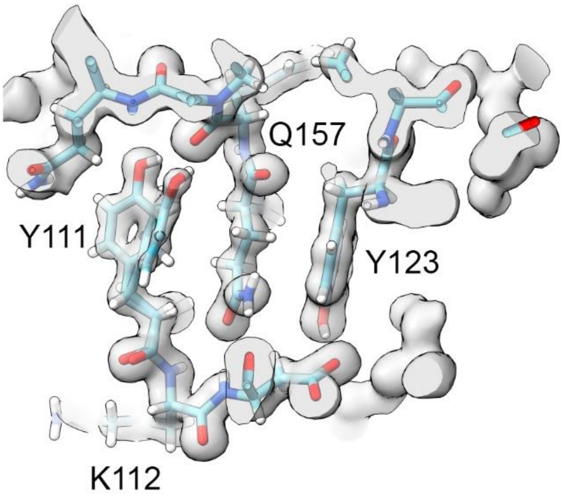

Supplementary Figure 2. Residues in the loop 2 with corresponding 2Fo-Fc map displayed at a contour level of about 1.2σ showing the double conformation of Y111 and with Q157 sandwiched by a π-amide stacking between one of the two Y111 rotamer conformations and Y123.

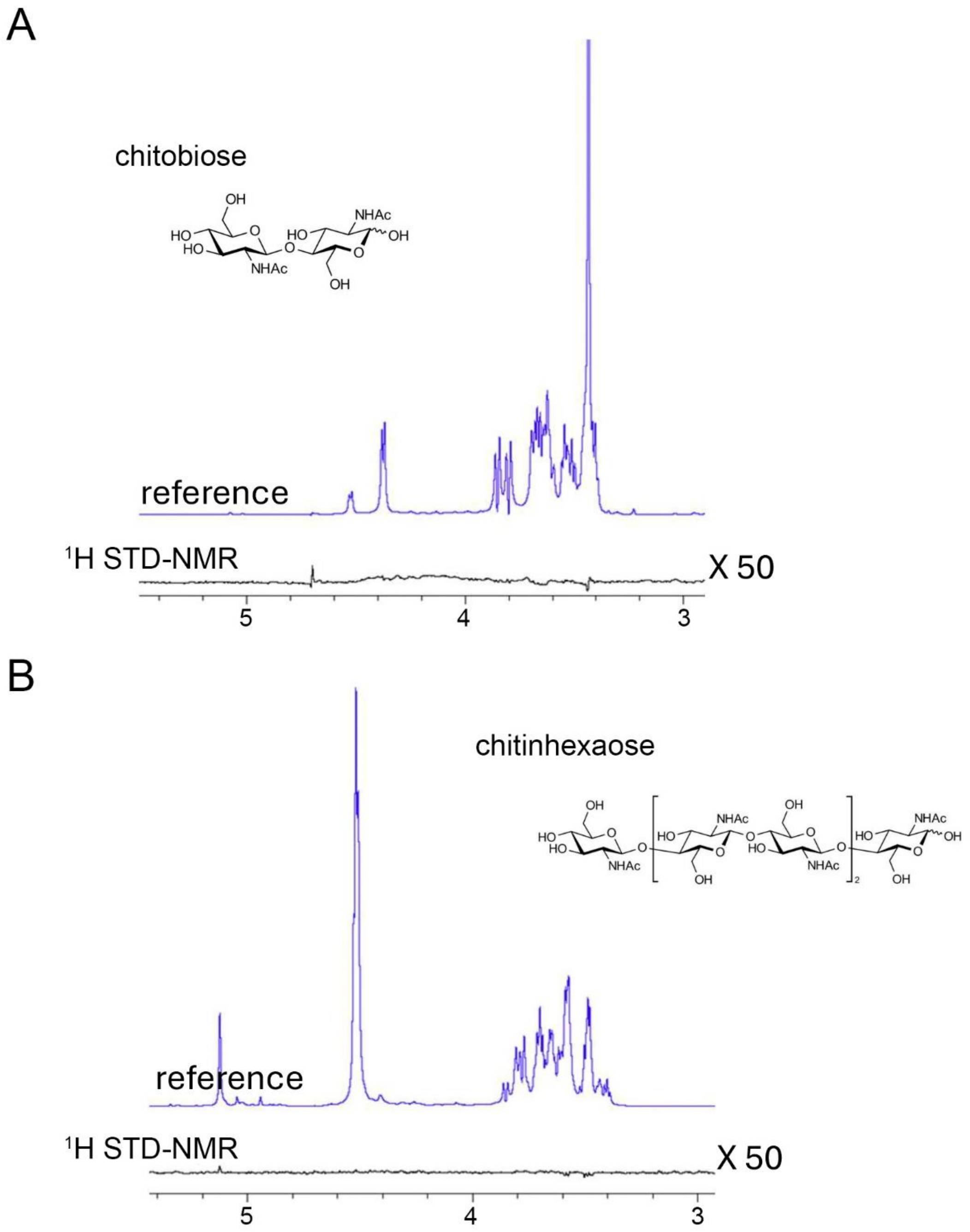

Supplementary Figure 3. (**A**) NMR results of chitiobiose binding to AgBR1. 1H-STD-NMR for a sample of 50µM AgBr1 and 80 equivalents of βOMeChitobiose: in blue, reference spectrum; in black, STD spectrum. (**B**) Same as (A) but using the chitinhexaose.

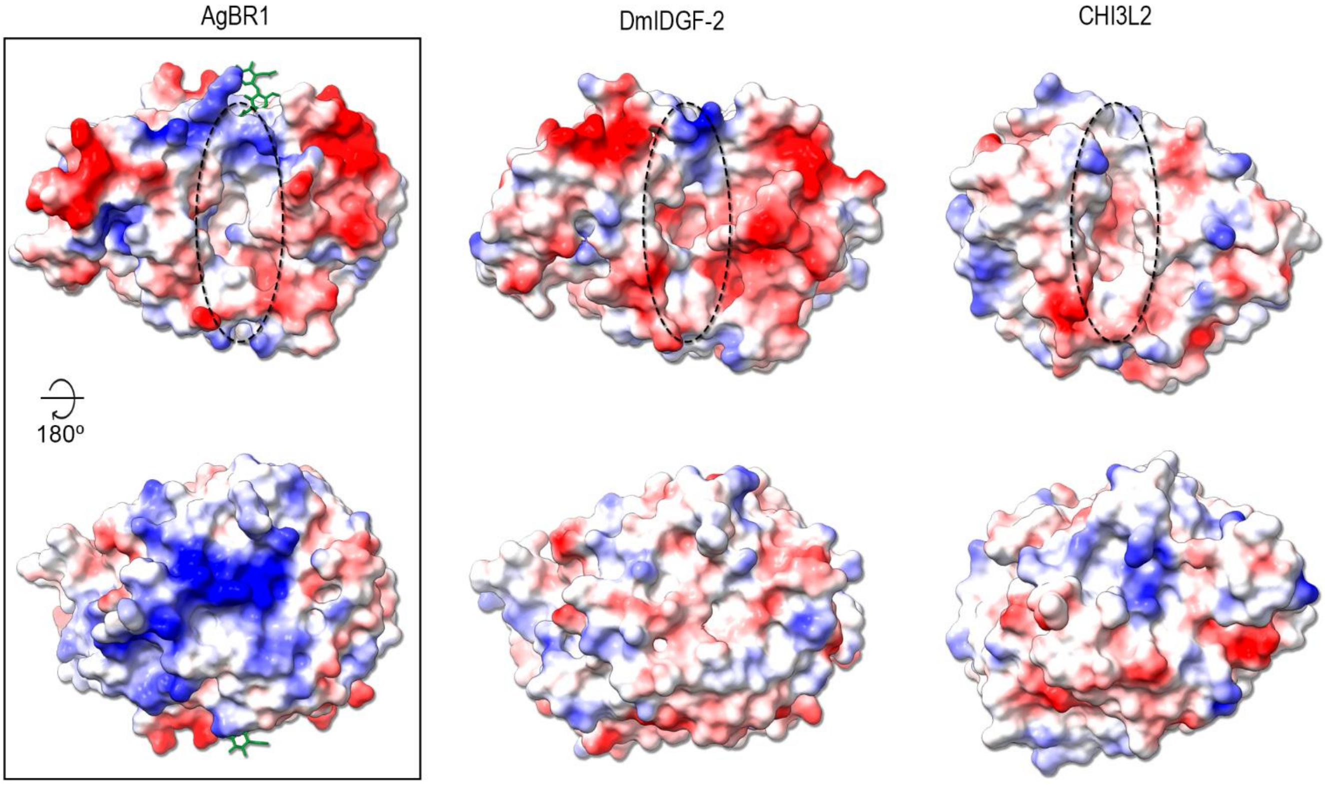

Supplementary Figure 4. Isopotential surface representation of AgBR1 (left), DmIDGF-2 (middle) and CHI3L2 (right). The electrostatic potential values range from 10 (blue) charge to -10 (red) measured in k_BT/e units. The black dashed lined oval localizes the putative ligand binding sites.

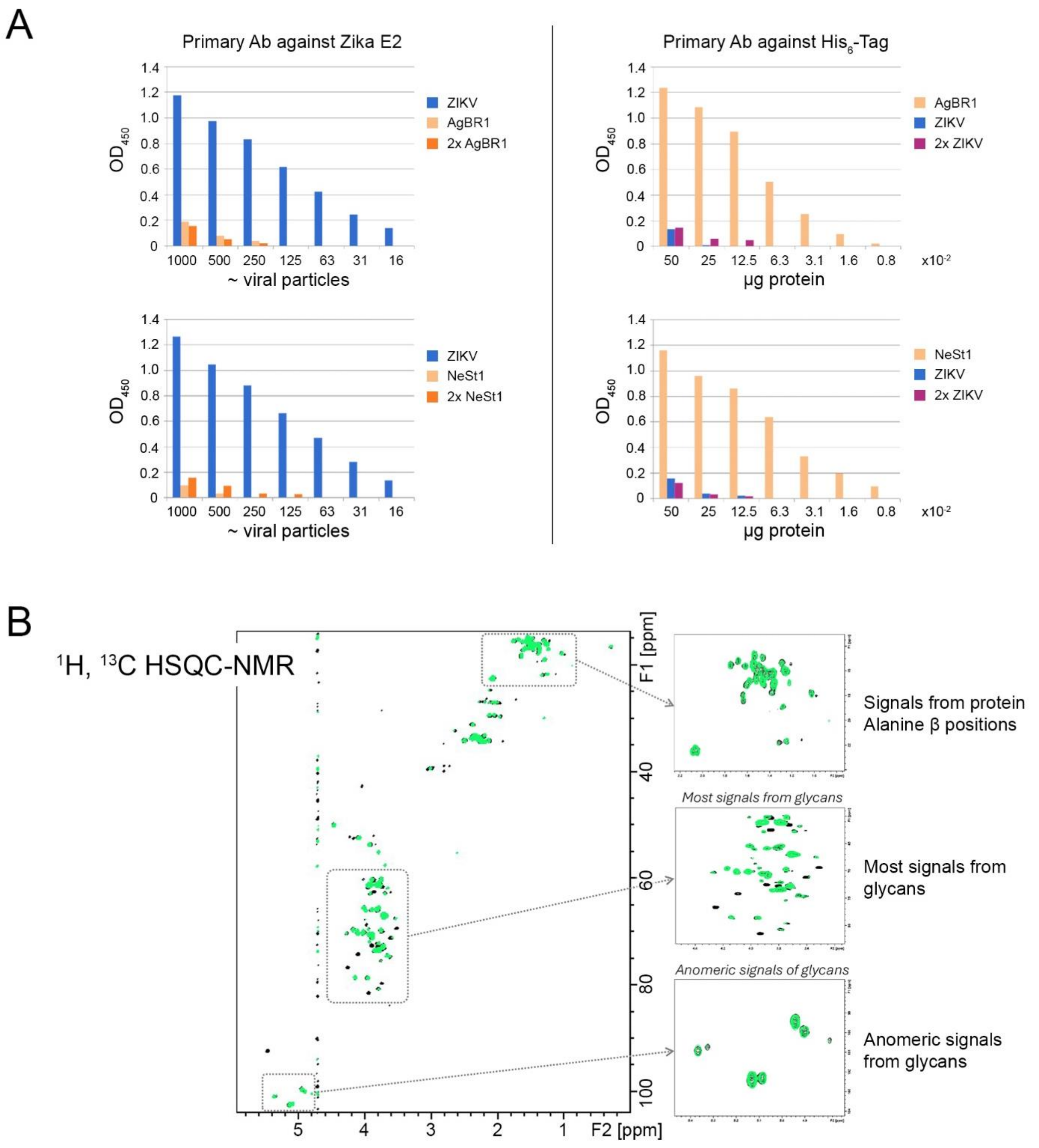

Supplementary Figure 5. (**A**) Histograms of fluorescence read-out from ELISA experiments when the whole virus is absorbed on the plate (left) and when AgBr1 or NeSt1 are absorbed on the plate (right). (**B**) NMR experiments of potential glycan-mediated binding of AgBR1 to Zika virus. Superimposition of 1H,13C-HSQC-NMR spectra of AgBr1 glycoprotein with 13C-glycans (overexpressed in HEK293): in black, control (without ZIKV) and in green with ZIKV. Insets show different zoomed regions of the spectrum. Only differences between the two spectra correspond to signals of sucrose.

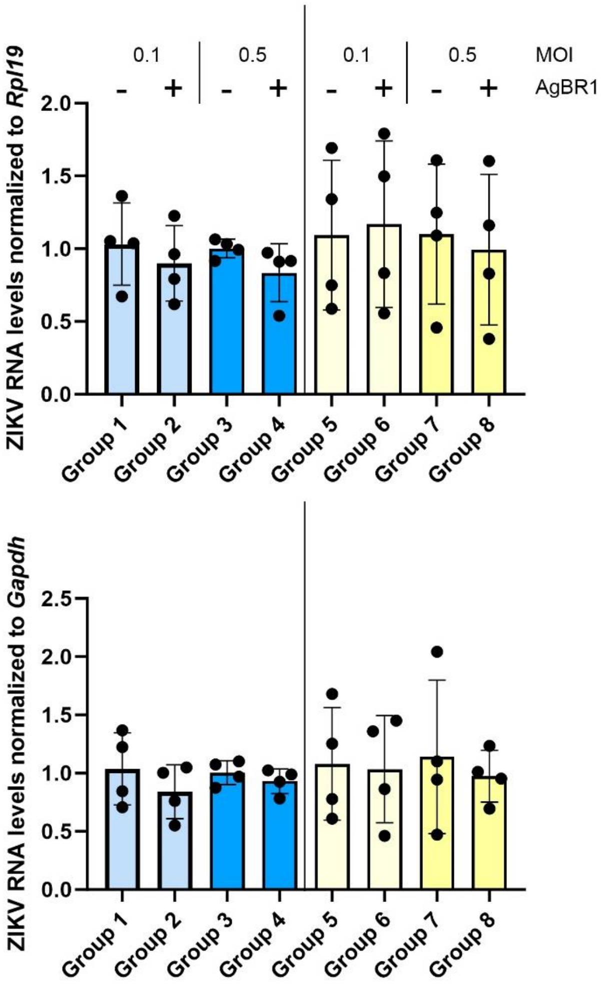

Supplementary Figure 6. ZIKV *glycoprotein E* gene RNA levels normalized either to *Rpol19* (top) or *Gapdh* (bottom) in BMDM cells infected at MOI 0.1 or 0.5 and in presence (+) or absence (-) of 5 µg of AgBR1 protein after 24 hours of incubation. The two independent experiments are colored in shades of blue and yellow. In experiment 1 (light and dark blue), Group 2 and Group 4 were compared to Group 1 and Group 3, respectively, and in experiment 2 (light and dark yellow), Group 6 and Group 8 were compared to Group 5 and Group 7, respectively. The mean mean ± SD refers to four independent biological replicates (BMDM cells derived from four independent mice). The gene expression fold-changes and p-values of considered comparisons are described in S1 Table.

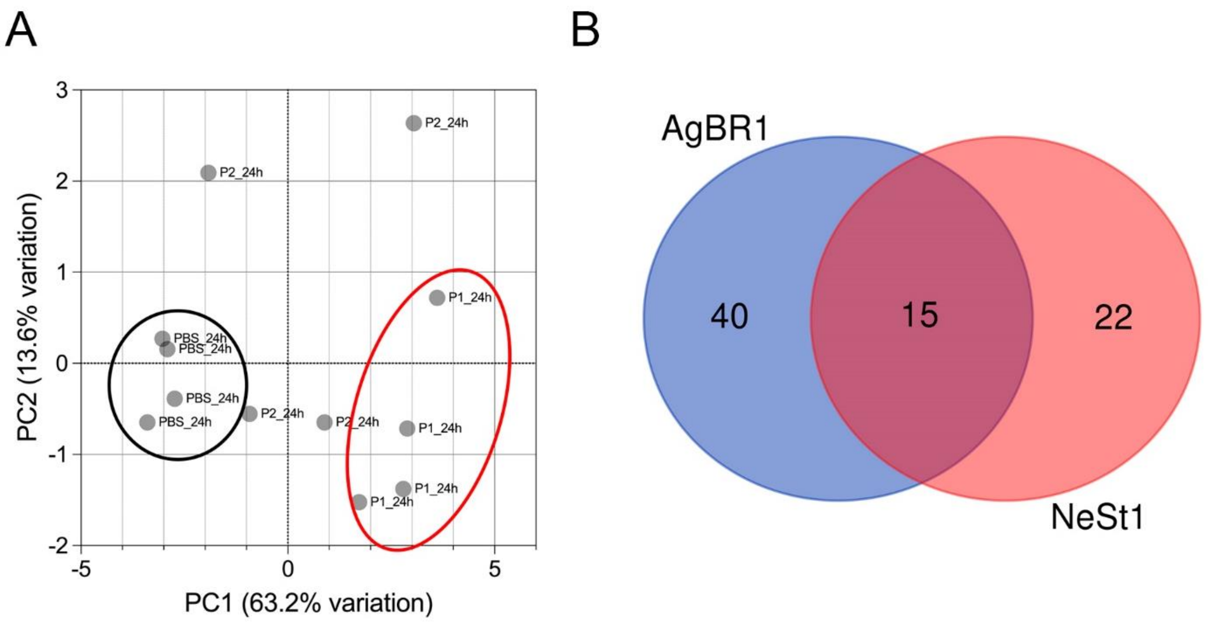

Supplementary Figure 7. (**A)** PCA analysis of protein samples at 24hrs of stimulation and unstimulated controls (P1, AgBR1 and P2, NeSt1). Blue and red circles mark segregation of control versus P1 experimental samples, respectively. (**B**) Venn diagram with the fifteen differentially expressed proteins in common between AgBR1 and NeSt1; Uniprot codes: Q8C878, Q3UV17, Q60710, P01899, Q01965, Q9JIW9, Q61549, Q9R233, P08103, P09671, P28667, P39688, P58058, P13597, and Q64345.

Supplementary Table 1. qPCR primer sequences, data and analysis

Supplementary Table 2. Uniprot accession codes of the over/under-expressed *Mus musculus* proteins in BMDM cells after 24 h of incubation with AgBR1. Only shown the differential proteins with a p-value < 0.05.

Supplementary Table 3. Uniprot accession codes of the over/under-expressed *Mus musculus* proteins in BMDM cells after 24 h of incubation with NeSt1. Only shown the differential proteins with a p-value < 0.05.

Supplementary Table 4. Biological processes identified by STRING for AgBR1 differentially expressed proteins in BMDM cells.

Supplementary Table 5. Biological processes identified by STRING for AgBR1 inside the predicted 'innate immune response' biological process.

Supplementary Table 6. Biological functions processes identified by STRING for NeSt1 differentially expressed proteins in BMDM cells.

Supplementary Table 7. Biological processes identified by STRING for NeSt1 within the predicted 'innate immune response' biological process.

Supplementary Table 8. Upstream regulators predicted by IPA for AgBR1 stimulation of BMDM cells.

Supplementary Table 9. Canonical pathways predicted by IPA for NeSt1 stimulation of BMDM cells.

Supplementary Table 10. Designed primers for AgBR1 and NeSt1 cloning and sequencing (5'-3' direction).

References

1. **Robert X, Gouet P.** 2014. Deciphering key features in protein structures with the new ENDscript server. Nucleic Acids Res. **42**(Web Server issue):W320-4.

2. **Varela PF, Llera AS, Mariuzza RA, Tormo J.** 2002. Crystal structure of imaginal disc growth factor-2. A member of a new family of growth-promoting glycoproteins from Drosophila melanogaster. J Biol Chem **277:**13229-13236.
